# OmpC-Dependent Bile Tolerance Contributes to E. coli Colonization of the Mammalian Intestine

**DOI:** 10.1128/spectrum.05241-22

**Published:** 2023-04-04

**Authors:** Sudhir Doranga, Tyrrell Conway

**Affiliations:** a Department of Microbiology and Molecular Genetics, Oklahoma State University, Stillwater, Oklahoma, USA; The Ohio State University Division of Biosciences

**Keywords:** bile tolerance, colonization, *Escherichia coli*, mammalian intestine, outer membrane proteins

## Abstract

Escherichia coli persistently colonizes the mammalian intestine by mechanisms that are not fully understood. Previously, we found when streptomycin-treated mice were fed E. coli MG1655, the intestine selected for *envZ* missense mutants that outcompeted the wild type. The better-colonizing *envZ* mutants had a higher level of OmpC and reduced OmpF. This suggested the EnvZ/OmpR two-component system and outer membrane proteins play a role in colonization. In this study, we show that wild-type E. coli MG1655 outcompetes an *envZ-ompR* knockout mutant. Moreover, *ompA* and *ompC* knockout mutants are outcompeted by the wild type, while an *ompF* knockout mutant colonizes better than the wild type. Outer membrane protein gels show the *ompF* mutant overproduces OmpC. An *ompC* mutant is more sensitive to bile salts than the wild type and *ompF* mutant. The *ompC* mutant initiates colonization slowly because it is sensitive to physiological concentrations of bile salts in the intestine. Overexpression of *ompC* under the control of a constitutive promoter confers a colonization advantage only when *ompF* is deleted. These results indicate that fine-tuning of OmpC and OmpF levels is needed to maximize competitive fitness in the intestine. RNA sequencing reveals the EnvZ/OmpR two-component system is active in the intestine: *ompC* is upregulated and *ompF* is downregulated. While other factors could also contribute to the advantage provided by OmpC, we provide evidence that OmpC is important for E. coli to colonize the intestine because its smaller pore size excludes bile salts or other unknown toxic substances, while OmpF is deleterious because its larger pore size allows bile salts or other unknown toxic substances to enter the periplasm.

**IMPORTANCE** Every mammalian intestine is colonized with Escherichia coli. Although E. coli is one of the most studied model organisms, how it colonizes the intestine is not fully understood. Here, we investigated the role of the EnvZ/OmpR two-component system and outer membrane proteins in colonization of the mouse intestine by E. coli. We report that an *ompC* mutant is a poor colonizer, while an *ompF* mutant, which overproduces OmpC, outcompetes the wild type. OmpF has a larger pore size that allows toxic bile salts or other toxic compounds into the cell and is deleterious for colonization of the intestine. OmpC has a smaller pore size and excludes bile salts. Our findings provide insights into why E. coli fine-tunes the levels of OmpC and OmpF during colonization via the EnvZ/OmpR two-component system.

## INTRODUCTION

We previously demonstrated that when the wild-type (WT) strain of Escherichia coli MG1655 was fed to streptomycin-treated mice, the intestine selected for mutants that grew faster on several carbon sources (1). Approximately 90% of the mutants were nonmotile, as a result of having 400- to 500-bp deletions in the promoter region of the *flhDC* operon that encodes the master regulator of the flagellar regulon. Deletions in the *flhDC* operon provided a competitive advantage in two ways: several catabolic pathways were upregulated, leading to more rapid growth, and energy conserved by being nonmotile was directed to other cellular activities (2, 3).

The remaining 10% of isolates retained motility yet outcompeted the wild type in competitive colonization assays (3). We subsequently showed the motile isolates were *envZ* missense mutants. One *envZ* mutant (P41L) was more resistant to colicin V and bile salts than the parental strain and grew faster on several sugars, suggesting that it gained an advantage in an intestinal niche not occupied by the parental strain (1). In a previous study, an *envZ* mutant (P41L) was shown to hyperphosphorylate OmpR (4). We subsequently showed that the motile mutant isolated from mice also had higher levels of OmpR~P than the wild type (5). In response to changing osmolarity, the response regulator OmpR of the EnvZ/OmpR two-component system regulates over 100 genes, including those encoding outer membrane proteins OmpC and OmpF (6). As expected, the E. coli MG1655 *envZ*_P41L_ mutant had a different outer membrane protein profile than the wild type (5). When the *envZ*_P41L_ gene was transferred from the E. coli MG1655 background into E. coli Nissle 1917, the newly constructed E. coli Nissle 1917 *envZ*_P41L_ strain outcompeted wild-type E. coli Nissle 1917 in the streptomycin-treated mouse intestine. E. coli Nissle 1917 *envZ*_P41L_ had higher levels of OmpR~P and displayed a different outer membrane protein profile than the wild type. Even at low osmolarity, Nissle 1917 *envZ*_P41L_ had a larger amount of OmpC and less OmpF than wild-type E. coli Nissle 1917 (5). Similar results were obtained when Giroud et al. fed E. coli MG1655 to germfree mice (7). The germfree mouse intestine selected for mutants in the EnvZ/OmpR two-component system, which repressed OmpF production and increased OmpC levels compared to the wild type, altering the outer membrane protein composition (7). These results suggested a possible role for outer membrane proteins in colonization. However, the importance of outer membrane proteins in colonization has not been studied.

Since the *envZ*_P41L_ mutants had higher OmpC and lower OmpF and were also more resistant to bile salts than the wild type (5, 7, 8), we hypothesized that OmpC is important for bile tolerance in the intestine. Compared to OmpC, OmpF forms a slightly larger pore, about 1.2 nm in diameter (9). Because of OmpF’s larger pore size, it allows almost 10-fold-faster diffusion of molecules, including bile salts, compared to the OmpC porin (10). Since bile salt is present in the intestine (11–13), it is expected that OmpF is detrimental to the bacteria, so they switch on OmpC and decrease OmpF expression (14). E. coli O157:H7 grown *in vitro* with 0.15% and 0.8% bile salts had 2.54- and 7-fold-lower *ompF* mRNA levels than untreated cultures, respectively (14, 15). Similarly, *micF* mRNA, which negatively regulates *ompF* expression, was upregulated, consistent with OmpF being detrimental when bile salt is present (14). However, *ompC* was not upregulated in either of those bile salt treatments (14, 15). Salmonella enterica serovar Typhi *ompC* mutants had a severe growth defect in LB medium supplemented with 5% bile salt compared with the wild type. However, an *ompF* mutant grew as well as the wild type in LB medium with 5% bile salts. When an *ompC* mutant was complemented, it restored growth in the presence of bile salts, which suggests that *Salmonella* Typhi requires OmpC porin, but not OmpF, to survive the detrimental effects of bile salts in the gut (16). This led us to hypothesize that OmpC, but not OmpF, is important for E. coli colonization of the mammalian intestine by allowing it to tolerate the physiological bile salt concentration.

Here, we present the findings that envZ-*ompR*, *ompA*, and *ompC* knockout mutants are outcompeted by the wild type during competition, whereas an *ompF* mutant is a better colonizer than the wild type. We found that *ompF* mutant competes with wild type in the same niche and that the *ompF* mutant does not have an advantage in nutrient utilization. Higher production of OmpC in the *ompF* mutant makes it a better colonizer; however, higher production of OmpC alone is not sufficient for better colonization, indicating the ratio of OmpC to OmpF is crucial. We also showed that an *ompC* mutant is a poor competitor because it takes time to adapt to the intestine. We confirmed, through transcriptome sequencing (RNA-seq) analysis of E. coli mRNA from mouse cecal mucus, that EnvZ/OmpR-dependent genes are regulated in the intestine, including *ompC*, which is upregulated by a log_2_ fold change of 1.73, while *ompF* is highly downregulated (log_2_ fold change of −5.56).

## RESULTS

### EnvZ/OmpR two-component system is important for E. coli MG1655 competition in the mammalian intestine.

**FIG 1:**
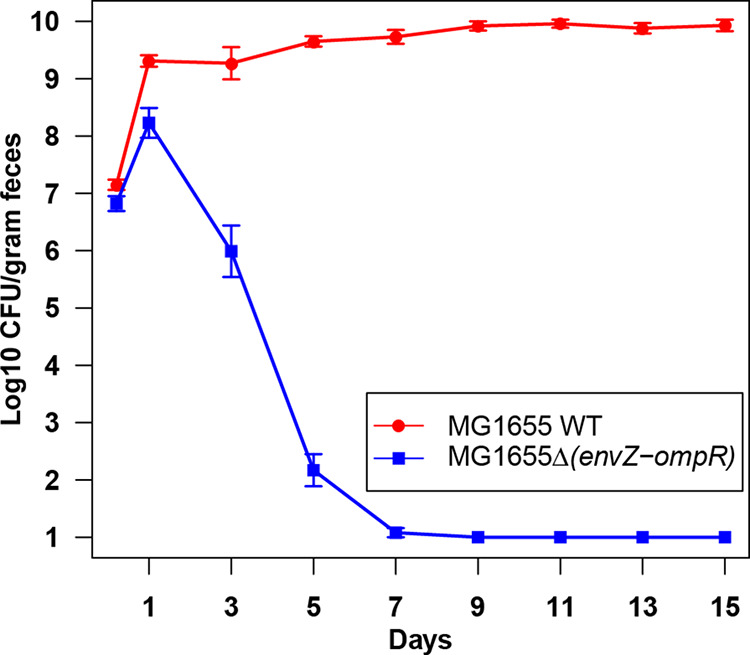
Colonization of the mouse intestine by the E. coli MG1655 Str^r^ Nal^r^ wild type and E. coli MG1655 Str^r^ Δ(*envZ-ompR*)*::cam* mutant. Two sets of three CD-1 male mice were fed 10^5^ CFU of the E. coli MG1655 Str^r^ Nal^r^ wild type and 10^5^ CFU of E. coli MG1655 Str^r^ Δ(*envZ-ompR*)::*cam*. At indicated times, samples were collected, homogenized, diluted, and plated. Error bars represent standard errors of the pooled data of log_10_ mean CFU per gram of feces.

To determine whether the EnvZ/OmpR two-component system has a role in colonization, we constructed the E. coli MG1655 Str^r^ Δ(*envZ-ompR*)::*cam* mutant by deleting the *ompB* locus. E. coli MG1655 Str^r^ Δ(*envZ-ompR*)::*cam* colonized the streptomycin-treated mouse intestine at ~10^8^ CFU/g of feces, which indicates the mutant colonizes well when it is the only E. coli strain in the intestine (see Fig. S1 in the supplemental material). However, when the mutant was competed against E. coli MG1655 Str^r^ Nal^r^ (wild type), the mutant was severely defective in competition and was eliminated completely within 9 days of competition (Fig. 1). This result indicates the EnvZ/OmpR two-component system is important for colonization.

### OmpC and OmpA are important while OmpF is detrimental for competition during colonization of the intestine.

We previously showed the *E coli* MG1655 *envZ*_P41L_ mutant strain had elevated levels of OmpC and lower levels of OmpF. Furthermore, *E coli* MG1655 *envZ*_P41L_ outcompeted the wild type during competition in the streptomycin-treated mouse intestine (1, 5). These results suggested that outer membrane proteins are important for colonization.

**FIG 2:**
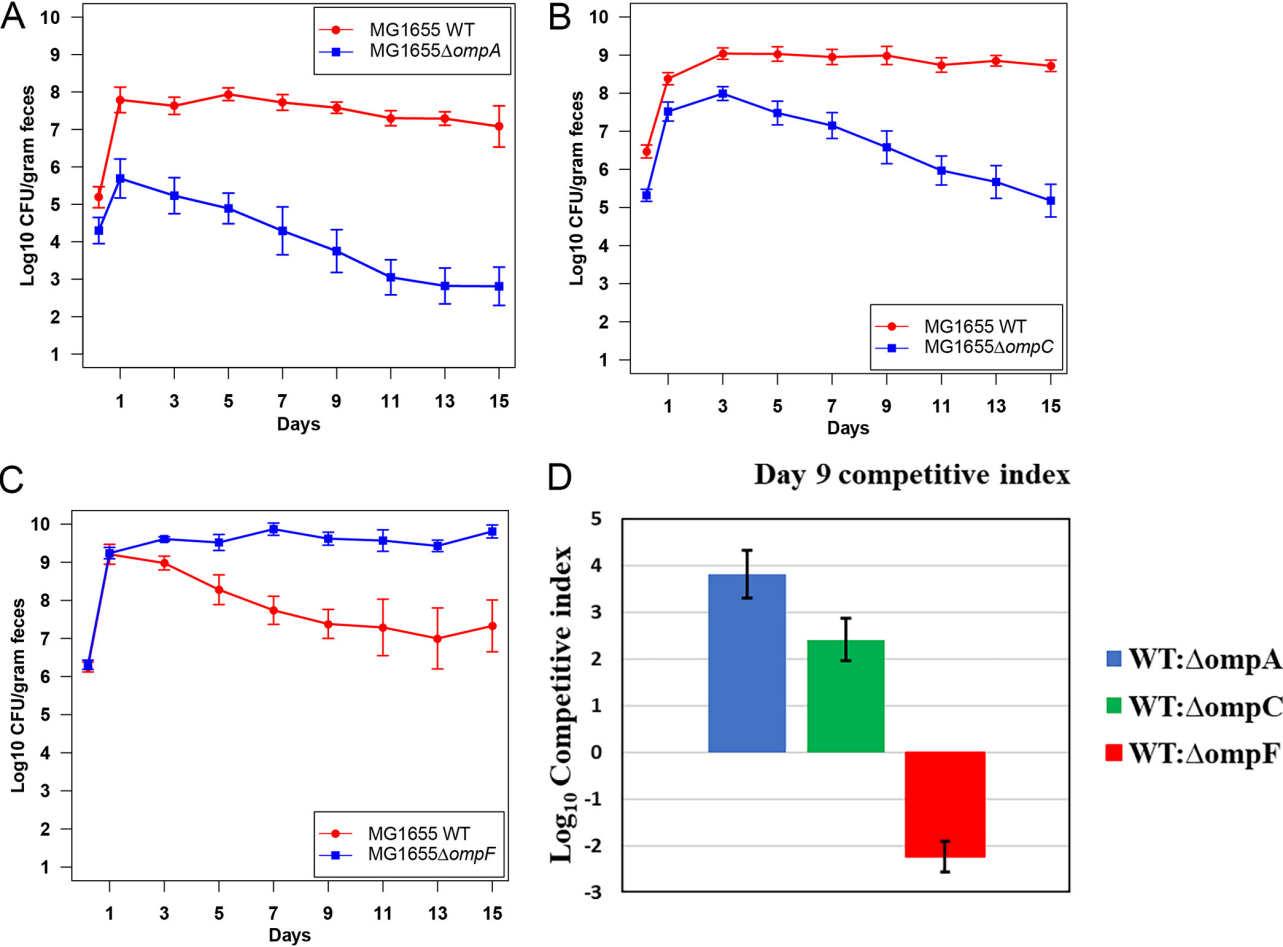
Colonization of the mouse intestine by the E. coli MG1655 Str^r^ Nal^r^ wild type and outer membrane protein mutants. (A) Two sets of three CD-1 male mice were fed 10^5^ CFU of the E. coli MG1655 Str^r^ Nal^r^ wild type and 10^5^ CFU of E. coli MG1655 Str^r^ Δ*ompA*::*cam*. (B) Four sets of three CD-1 male mice were fed 10^5^ CFU of the E. coli MG1655 Str^r^ Nal^r^ wild type and 10^5^ CFU of E. coli MG1655 Str^r^ Δ*ompC*::*cam*. (C) Two sets of three CD-1 male mice were fed 10^5^ CFU of the E. coli MG1655 Str^r^ Nal^r^ wild type and 10^5^ CFU of E. coli MG1655 Str^r^ Δ*ompF*::*cam.* At indicated times, samples were collected, homogenized, diluted, and plated. Error bars represent standard errors of the pooled data of log_10_ mean CFU per gram of feces. (D) Log_10_ competitive index of WT versus mutant at 9 days of competition.

To determine the role of outer membrane proteins in colonization, we focused on OmpA, OmpC, and OmpF, two of which (OmpC and OmpF) are regulated by the EnvZ/OmpR two-component system. We constructed knockout mutants of *ompA*, *ompC*, and *ompF* and fed them to streptomycin-treated mice. All three mutants colonized equally well at ~10^9^ CFU/g of feces, indicating the mutants can colonize the mammalian intestine when fed alone (Fig. S2A, B, and C). Next, each mutant was competed with the E. coli MG1655 Str^r^ Nal^r^ wild type for colonization of the mouse intestine. E. coli MG1655 Str^r^ Δ*ompA*::*cam* and E. coli MG1655 Str^r^ Δ*ompC*::*cam* mutants were outcompeted by the E. coli MG1655 Str^r^ Nal^r^ wild type and demonstrated colonization defects in initiation (24 h) and maintenance (day 9) (Fig. 2A and B). At day 9 postfeeding, the log_10_ competitive indexes for the wild type versus E. coli MG1655 Str^r^ Δ*ompA*::*cam* and wild type versus E. coli MG1655 Str^r^ Δ*ompC*::*cam* were 3.82 and 2.41, respectively (Fig. 2D). In contrast, E. coli MG1655 Str^r^ Δ*ompF*::*cam* outcompeted the wild type during colonization (Fig. 2C). At 9 days postfeeding, the log_10_ competitive index for the wild type versus E. coli MG1655 Str^r^ Δ*ompF*::*cam* was −2.24 (Fig. 2D). In our experience, gain-of-function mutations that increase the capacity for colonization are rare in E. coli. Only nonmotile Δ*flhDC* mutants and motile *envZ* missense mutants have been found previously to colonize better than the wild type (1, 2). The E. coli MG1655 Str^r^ Δ*flhDC* mutants grew significantly faster than their parent strains on several sugars that have been previously identified as being important for colonization (2). The E. coli MG1655 Str^r^ *envZ* missense mutant grew ~30% faster than E. coli MG1655 Str^r^ Nal^r^ on galactose *in vitro* and used galactose to colonize a smaller distinct intestinal niche (1). This led us to test whether E. coli MG1655 Δ*ompF*::*cam* competes in a different niche than the wild type or if it competes in the same niche.

### Δ*ompA* and Δ*ompF* mutants compete with the wild type in the same niche, while an Δ*ompC* mutant fails to saturate a minor niche.

**FIG 3:**
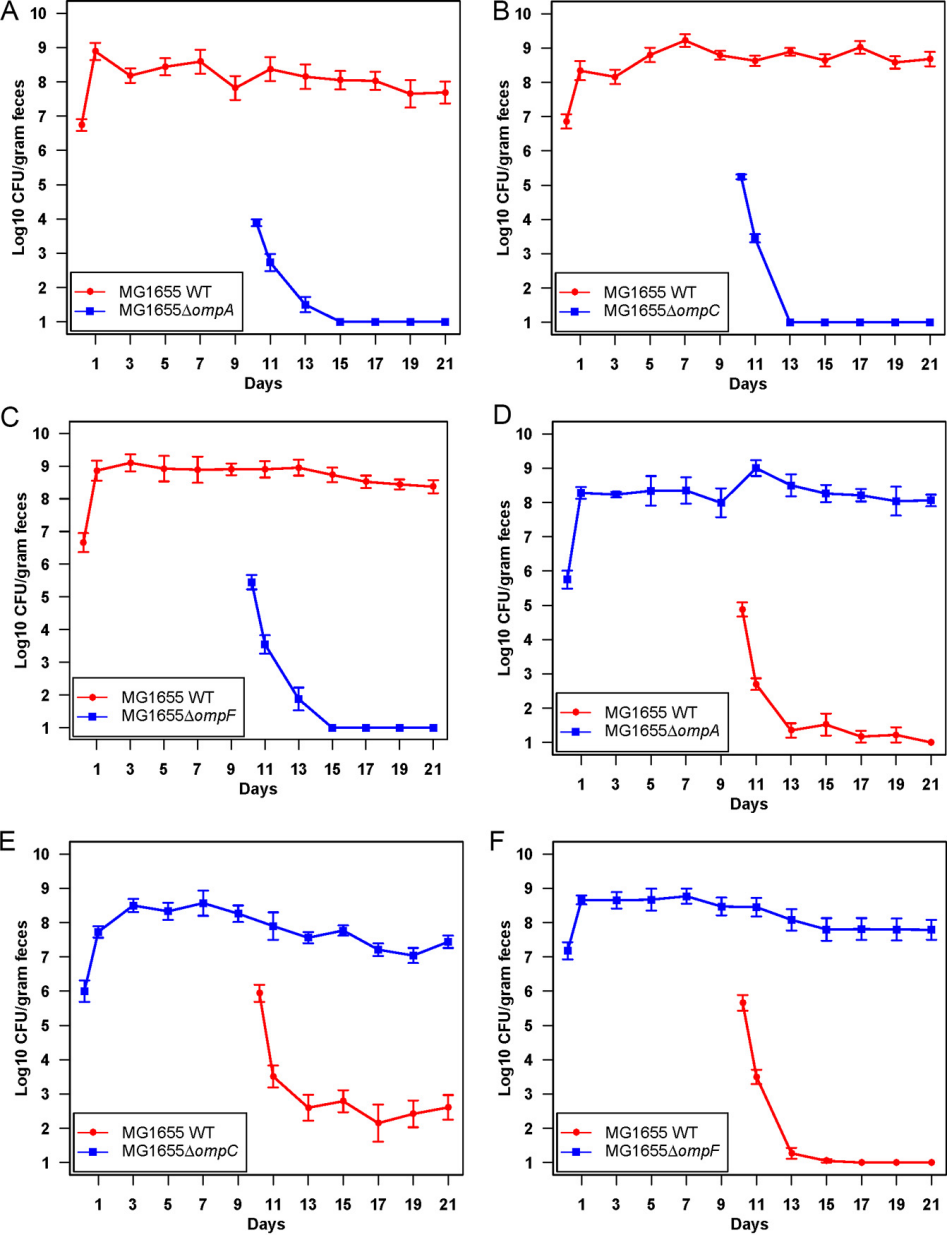
Competitive colonization of the E. coli MG1655 Str^r^ Nal^r^ wild type and outer membrane protein mutants. Two sets of 3 mice were fed 10^5^ CFU of E. coli MG1655 Str^r^ Nal^r^ wild type and 10 days later were fed 10^5^ CFU of (A) E. coli MG1655 Str^r^ Δ*ompA*::*cam*, (B) E. coli MG1655 Str^r^ Δ*ompC*::*cam*, or (C) E. coli MG1655 Str^r^ Δ*ompF*::*cam*. In reverse experiments, two sets of 3 mice were fed 10^5^ CFU of (D) E. coli MG1655 Str^r^ Δ*ompA*::*cam*, (E) E. coli MG1655 Str^r^ Δ*ompC*::*cam*, or (F) E. coli MG1655 Str^r^ Δ*ompF*::*cam* and 10 days later were fed 10^5^ CFU of the E. coli MG1655 Str^r^ Nal^r^ wild type.

To test whether or not E. coli MG1655 Str^r^ **Δ***ompF*::*cam* occupies a distinct niche in the intestine, we precolonized streptomycin-treated mice with the E. coli MG1655 Str^r^ Nal^r^ wild type by feeding ~10^5^ CFU, which grew to ~10^9^ CFU/g of feces. On the 10th day of the experiment, the precolonized mice were fed 10^5^ CFU of E. coli MG1655 Str^r^ **Δ***ompA*::*cam*, E. coli MG1655 Str^r^ **Δ***ompC*::*cam*, or E. coli MG1655 Str^r^ **Δ***ompF*::*cam*. All three mutants failed to grow to high numbers in competition with the wild type and were eliminated within a few days of feeding (Fig. 3A to C). These results suggest that none of the *omp* mutants, including E. coli MG1655 Str^r^ **Δ***ompF*::*cam*, occupies a distinct niche in the intestine. In the reciprocal experiments, mice were precolonized with E. coli MG1655 Str^r^ **Δ***ompA*::*cam*, E. coli MG1655 Str^r^ **Δ***ompC*::*cam*, or E. coli MG1655 Str^r^ **Δ***ompF*::*cam* and then challenged with E. coli MG1655 Str^r^ Nal^r^ wild type. The E. coli MG1655 Str^r^ Nal^r^ wild type was eventually eliminated (21st day of the experiment) from mice precolonized with E. coli MG1655 Str^r^ **Δ***ompA*::*cam* (Fig. 3D). Similarly, in mice precolonized with E. coli MG1655 Str^r^ **Δ***ompF*::*cam*, the wild type was eliminated rapidly, within 3 days of the challenge (Fig. 3F). This suggests that the wild type competes in the same niche as E. coli MG1655 Str^r^ **Δ***ompA*::*cam-* and E. coli MG1655 Str^r^ **Δ***ompF*::*cam*-precolonized mice.

However, the E. coli MG1655 Str^r^ Nal^r^ wild type was not eliminated from E. coli MG1655 Str^r^ **Δ***ompC*::*cam*-precolonized mice and persisted at 10^2^ to 10^3^ CFU/g of feces (Fig. 3E). This suggests E. coli MG1655 Str^r^ **Δ***ompC*::*cam* fails to saturate a minor niche in which the wild type can grow. To determine if the minor niche is defined by nutrient consumption, we grew E. coli MG1655 Str^r^ **Δ***ompC*::*cam*, E. coli MG1655 Str^r^ **Δ***ompF*::*cam*, and the E. coli MG1655 Str^r^ Nal^r^ wild type individually in MOPS (morpholinepropanesulfonic acid) medium containing 9 different sugars (0.2% [wt/vol]). The wild type and mutants grew at approximately the same rate on all sugars (Fig. S3A to I), suggesting that E. coli MG1655 Str^r^ **Δ***ompC*::*cam* does not fail to saturate any of these sugar niches. Taken together, these results suggest that *ompA* and *ompF* mutants compete with the wild type in the same intestinal niche, while the *ompC* mutant fails to saturate a minor, unknown intestinal niche.

### Production of OmpC is crucial for better colonization by E. coli MG1655 Δ*ompF*.

**FIG 4:**
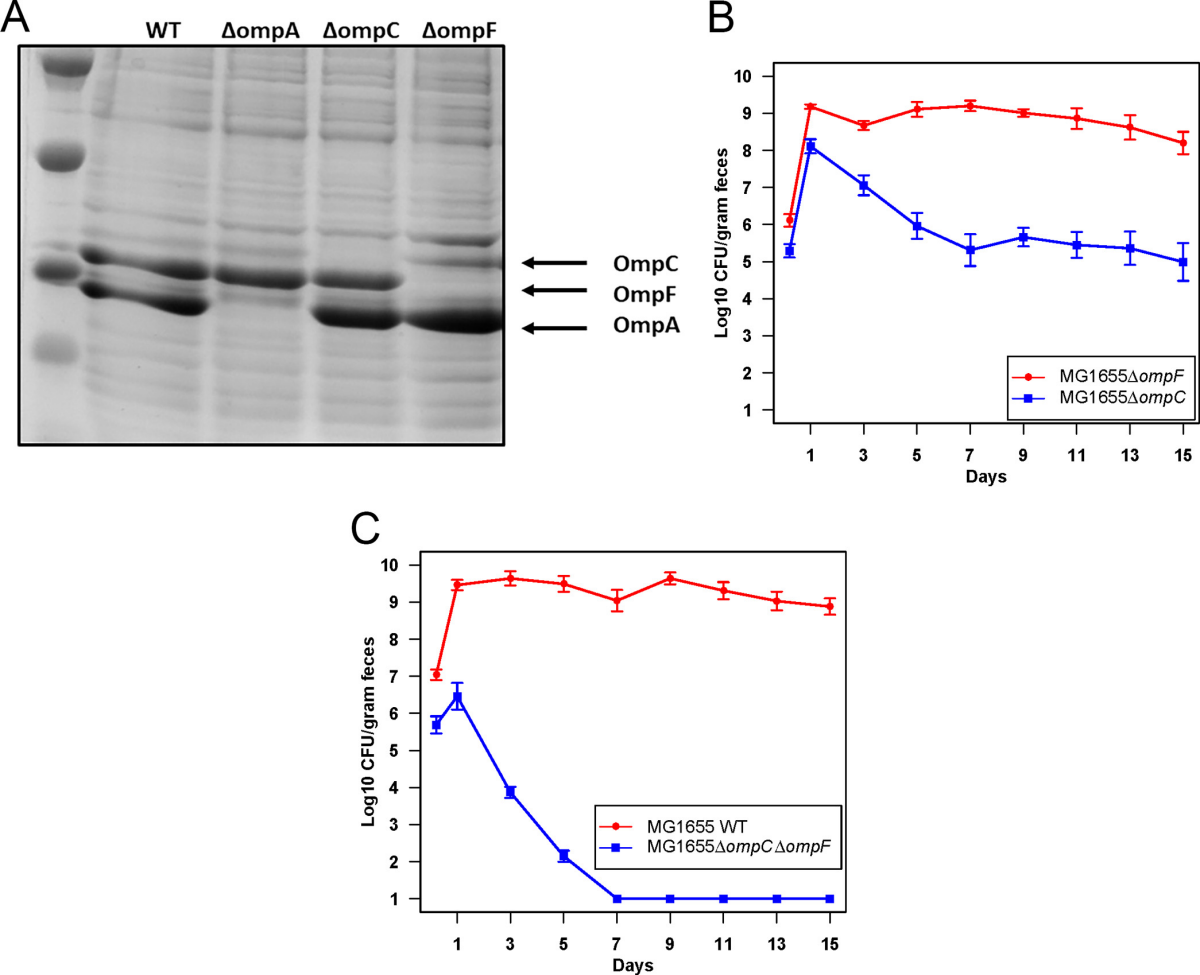
Importance of OmpC in E. coli colonization of the mouse intestine. (A) OmpA, OmpC, and OmpF levels in the outer membranes of the E. coli MG1655 Str^r^ Nal^r^ wild type and outer membrane protein mutants grown in low-osmolarity medium. Densitometric analysis is shown in Fig. S4. (B) Competitive colonization of the mouse intestine by the E. coli MG1655 Str^r^ Δ*ompF*::*cam* and E. coli MG1655 Str^r^ Δ*ompC*::*kan* mutants; (C) competitive colonization of the mouse intestine by the E. coli MG1655 Str^r^ Nal^r^ wild type and E. coli MG1655 Str^r^ Cam^r^ Δ*ompC* Δ*ompF* mutant.

Since E. coli MG1655 Str^r^ **Δ***ompF*::*cam* competes with the wild type in the same niche, we sought to determine why the *ompF* mutant has a competitive advantage. Outer membrane porins OmpC and OmpF are under tight regulation by the EnvZ/OmpR two-component system (17). At high osmolarity, EnvZ phosphorylates OmpR and OmpR~P binds to the promoter and activates *ompC*. At low osmolarity, high phosphatase activity of EnvZ reduces the level of OmpR~P, which binds to the promoter of *ompF* to activate its expression (18). Although the relative levels of these two outer membrane proteins vary under different environmental conditions, such as temperature, osmolarity, carbon source, etc., the collective amount of these proteins remains constant (19). Previously, it was shown that an E. coli K-12 strain having a chromosomal deletion beginning upstream of *ompC* and extending through the *ompC* gene was constitutive for OmpF production (20). Likewise, when an *ompF* mutation was introduced into E. coli H0201 by selecting for resistance to phage TuIa (OmpF acts as a receptor for phage TuIa), OmpC protein was made at a fully induced level even in low-osmolarity medium (21). This supports previous findings that when there is change in the level of one protein, the other protein is reciprocally regulated to compensate (21). This led us to predict that E. coli MG1655 Str^r^ Δ*ompF*::*cam* produces a larger amount of OmpC, which could contribute to better colonization. Outer membrane proteins of the E. coli MG1655 Str^r^ Nal^r^ wild type, E. coli MG1655 Str^r^ Δ*ompA*::*cam*, E. coli MG1655 Str^r^ Δ*ompC*::*cam*, and E. coli MG1655 Str^r^ Δ*ompF*::*cam* grown in low-osmolarity medium (medium A) were extracted and analyzed by SDS-PAGE. We found that E. coli MG1655 Str^r^ Δ*ompF*::*cam* produced higher OmpC protein levels (5-fold higher) than the wild type (Fig. 4A; and Fig. S4). To test the importance of OmpC for colonization, E. coli MG1655 Str^r^ Δ*ompC*::*kan* was competed against E. coli MG1655 Str^r^ Δ*ompF*::*cam* in streptomycin-treated mice. E. coli MG1655 Str^r^ Δ*ompC*::*kan* was outcompeted by E. coli MG1655 Str^r^ Δ*ompF*::*cam* by almost 4 orders of magnitude (Fig. 4B). Moreover, when MG1655 Str^r^ Cam^r^ Δ*ompC* Δ*ompF* was competed against the wild type, it was eliminated within 7 days of competition (Fig. 4C). The MG1655 Str^r^ Cam^r^ Δ*ompC* Δ*ompF* double mutant can, however, colonize at wild-type levels when fed alone to mice (Fig. S5). These results suggest that overproduction of OmpC was crucial for better colonization of E. coli MG1655 Δ*ompF*::*cam*.

### OmpC overproduction alone is not sufficient for better colonization of the mammalian intestine.

**FIG 5:**
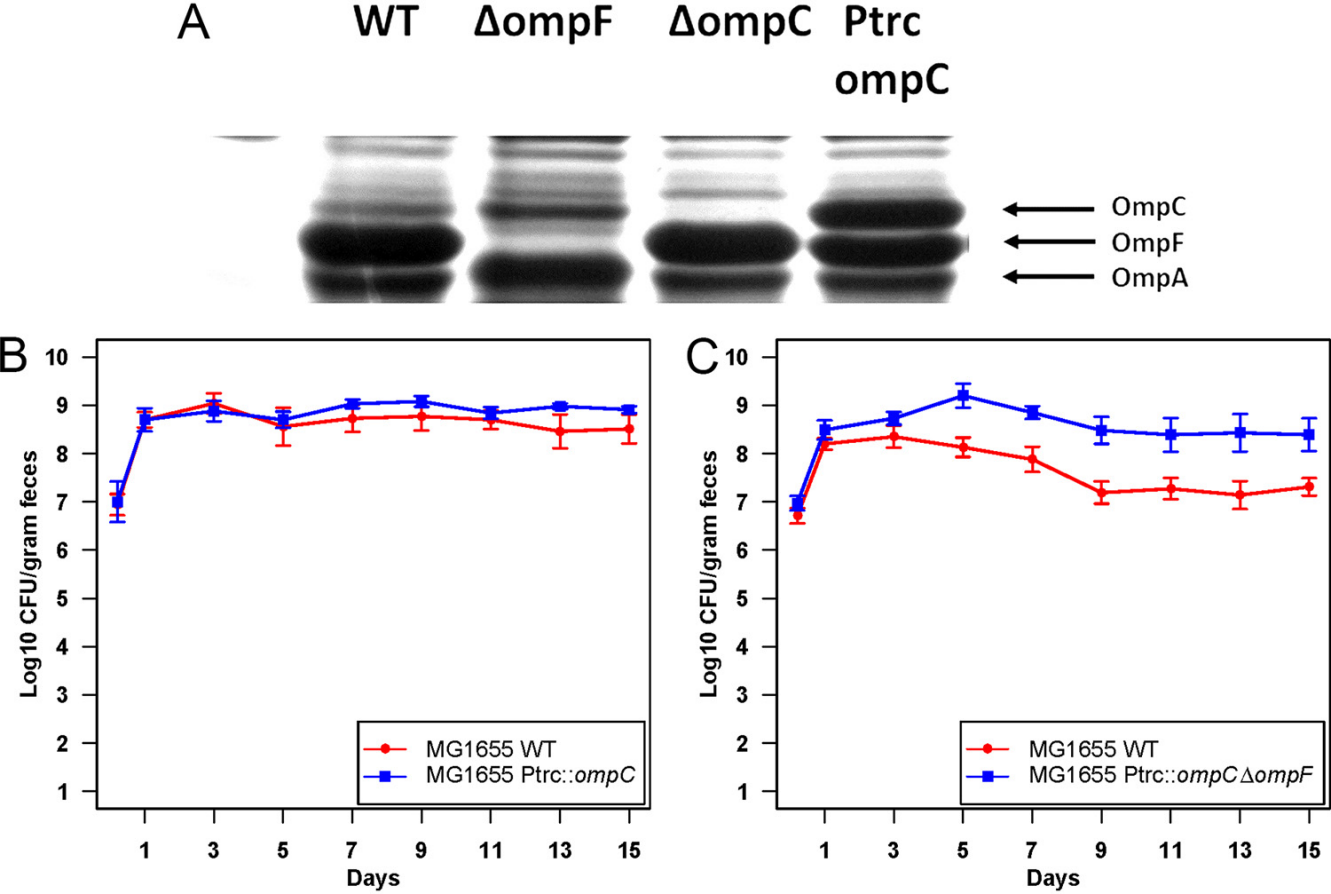
OmpC overproduction alone is not sufficient for better colonization of the mammalian intestine. (A) Outer membrane protein analysis showing overproduction of OmpC from P_trc_ promoter. Densitometric analysis is shown in Fig. S6. (B) Colonization of the mouse intestine by the E. coli MG1655 Str^r^ Nal^r^ wild type and E. coli MG1655 Str^r^ P*_trc_*::*ompC* mutant. (C) Colonization of the mouse intestine by the E. coli MG1655 Str^r^ Nal^r^ wild type and E. coli MG1655 Str^r^ Cam^r^ P_trc_::*ompC* Δ*ompF* mutant. At indicated times, samples were collected, homogenized, diluted and plated. Error bars represent standard errors of the pooled data of log_10_ mean CFU per gram of feces.

To determine whether OmpC overproduction alone is sufficient for better colonization, we constructed an OmpC-overproducing strain (E. coli MG1655 Str^r^ P*_trc_*::*ompC*) by replacing the regulatory region and the native promoter of *ompC* with a strong *trc* promoter (22) (Fig. 5A; Fig. S6). When E. coli MG1655 Str^r^ P*_trc_*::*ompC* was competed against the E. coli MG1655 Str^r^ Nal^r^ wild type, the two colonized equally well, suggesting that OmpC overproduction alone is not sufficient for better colonization (Fig. 5B). The presence of OmpF may interfere with the advantage conferred by constitutively overproduced OmpC. To test this possibility, we deleted *ompF* from the overproducing strain to generate E. coli MG1655 Str^r^ Cam^r^ P*_trc_*::*ompC* Δ*ompF*, which overproduces OmpC but does not produce OmpF. E. coli MG1655 Str^r^ Cam^r^ P*_trc_*::*ompC* Δ*ompF* colonized better during competition against the E. coli MG1655 Str^r^ Nal^r^ wild type (Fig. 5C), which indicates that fine-tuning of OmpC and OmpF production is important for colonization.

### An *ompC* mutant is defective in initiation of colonization.

**FIG 6:**
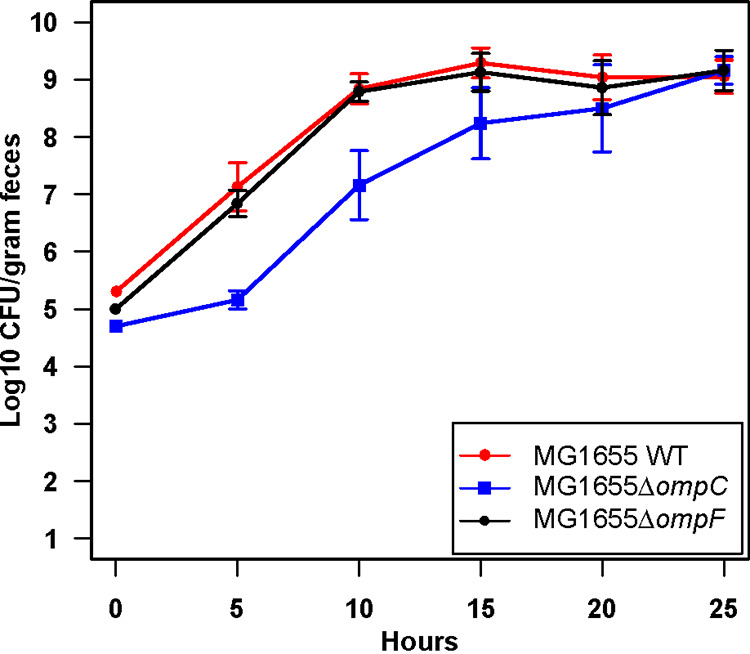
Monocolonization of the mouse intestine by E. coli MG1655 strains for 25 h. One set of 3 mice each were fed with 10^5^ CFU of the E. coli MG1655 Str^r^ Nal^r^ wild type, E. coli MG1655 Str^r^ Δ*ompC*::*cam* or MG1655 Str^r^ Δ*ompF*::*cam* mutant, and the fecal samples were collected at 5, 10, 15, 20, and 25 h. Time point 0 indicates CFU of strains fed to mice in 1 mL of 5% sucrose solution.

Next, we were interested in why E. coli MG1655 St^r^ Δ*ompC*::*cam* was outcompeted by its wild-type parent during competition. We noted in all cases when E. coli MG1655 Str^r^ Δ*ompC*::*cam* was competed in mice against the wild type or other *omp* mutants that the *ompC* mutant initiated colonization at a lower level, although approximately equal populations of both strains were fed to mice (Fig. 2B and Fig. 4). Therefore, we compared the populations of each strain at the time of feeding to the populations 5 h after feeding and found that E. coli MG1655 Str^r^ Δ*ompC*::*cam* initiated colonization more slowly than the wild type (Table 1). To more closely examine colonization initiation by E. coli MG1655 Str^r^ Δ*ompC*::*cam*, we fed ~10^5^ CFU of the *ompC* and *ompF* mutants or the wild type individually to streptomycin-treated mice and followed their populations at 5-h intervals for the first 25 h of colonization. We found that colonization by E. coli MG1655 Str^r^ Δ*ompC*::*cam* was slower than that of the *ompF* mutant and the wild type through the first 15 h, although the *ompC* mutant reached ~10^9^ CFU/g of feces by 25 h of colonization (Fig. 6). This result indicates that E. coli MG1655 Str^r^ Δ*ompC*::*cam* is defective in adapting to the intestine and lags during the initiation stage of colonization, which explains why it is outcompeted by the wild type.

**TABLE 1:** Populations of the *E. coli* MG1655 WT or Δ*ompC* mutant fed in experiments and recovered after 5 h from feces of mice

Expt	Log_10_ CFU/mL of MG1655 WT or Δ*ompC* mutant fed to mice	Log_10_ CFU/g of feces of MG1655 WT or mutant recovered after 5 h	Increase in log_10_ CFU in 5 h
MG1655 WT fed to mice and recovered			
MG1655 WT vs MG1655 Δ*ompC*	5.16	6.67	1.51
MG1655 WT vs MG1655 Δ*ompC* Δ*ompF*	5.30	7.04	1.74
MG1655 WT vs MG1655 P*_trc_*::ompC	4.90	6.94	2.04
MG1655 WT vs MG1655 P*_trc_*::ompC Δ*ompF*	5.18	6.71	1. 53
MG1655 Δ*ompC* mutant fed to mice and recovered			
MG1655 WT vs MG1655 Δ*ompC*	5.00	5.33	0.33
MG1655 WT vs MG1655 Δ*ompC* (E2)	4.93	5.33	0.4
MG1655 Δ*ompC*	5.50	5.03	−0.47
MG1655 Δ*ompF* vs MG1655 Δ *ompC*	5.18	5.29	0.11
MG1655 Δ*ompC* challenged by WT	5.40	6.00	0.6

### E. coli MG1655 Str^r^ Δ*ompC*::*cam* is sensitive to bile salts at a physiological concentration in the intestine and competes poorly with the wild type in the presence of bile salts *in vitro*.

Next, we determined if sensitivity to bile salts is the reason that E. coli MG1655 Str^r^ Δ*ompC*::*cam* is defective in colonization initiation. Liver cells synthesize bile acids and conjugate them to taurine or glycine to form bile salts, which are concentrated and stored in the gallbladder (23). Bile salts are then released into the duodenum upon stimulation by an incoming meal (23, 24). Bile salts aid in the digestion and absorption of fat in the small intestine, where a high concentration of bile salts is maintained (25). Eventually, bile salts are reabsorbed into the circulation in the distal ileum (23, 25). Depending upon the individual and the types of food consumed, the concentration of bile salts in the small intestine varies between 0.2 and 2.0% (wt/vol) (11, 12). Although most bile is reabsorbed in the small intestine, the concentrations in feces are ~0.1% on a low-fat diet and ~0.3% on a high-fat diet (13). This concentration of bile salts could cause E. coli MG1655 Str^r^ Δ*ompC*::*cam* to lag during colonization.

**FIG 7:**
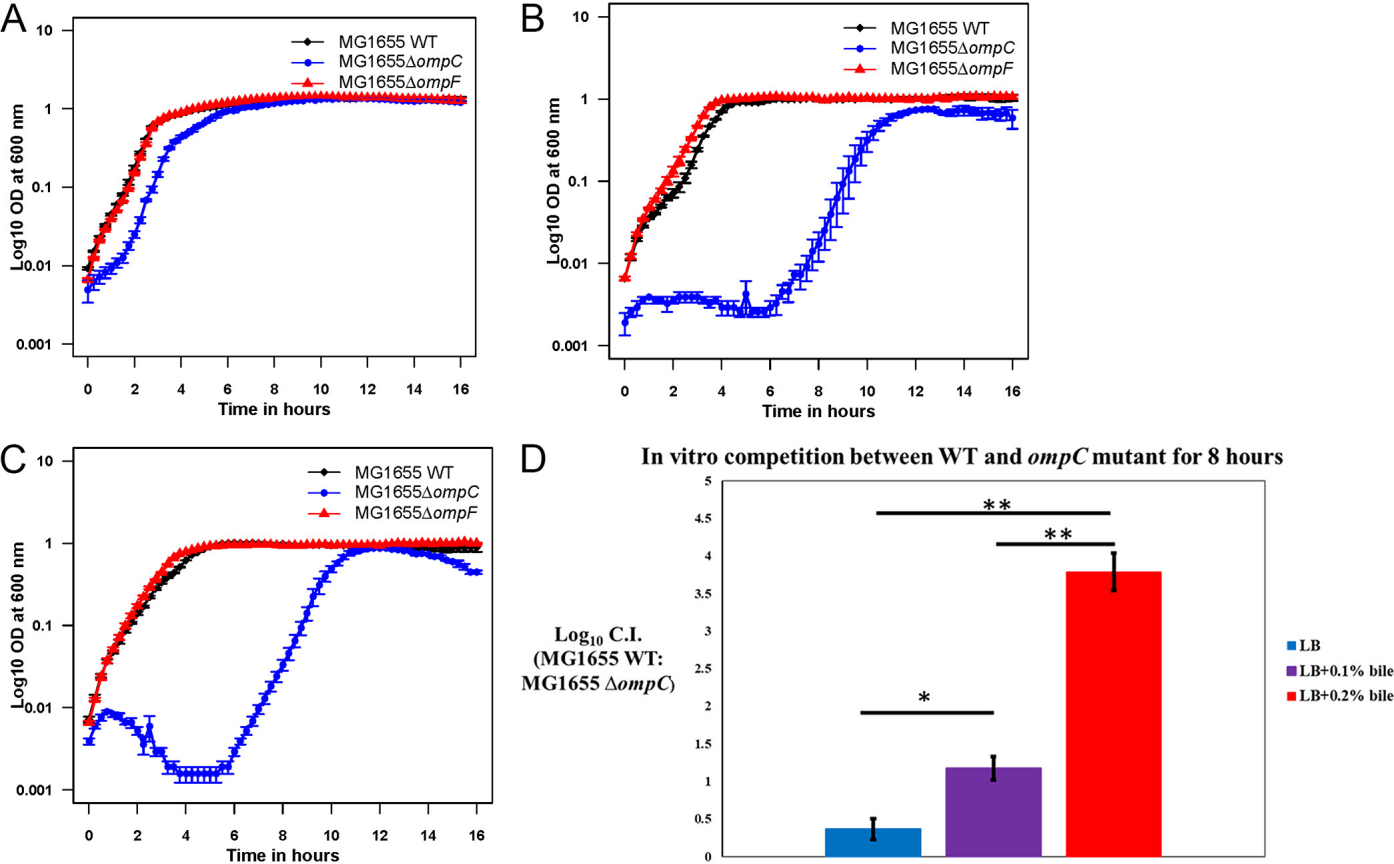
Growth of the E. coli MG1655 Str^r^ Nal^r^ wild type and E. coli MG1655 Str^r^ Δ*ompC*::*cam* and MG1655 Str^r^ Δ*ompF*::*cam* mutants in LB medium containing different concentration of bile salts. (A) Growth of E. coli strains in LB medium containing 0.1% bile salts; (B) growth of E. coli strains in LB medium containing 0.25% bile salts; (C) growth of E. coli strains in LB medium containing 0.5% bile salts; (D) *in vitro* competition between the E. coli MG1655 Str^r^ Nal^r^ wild type and E. coli MG1655 Str^r^ Δ*ompC*::*cam* mutant. The WT and the mutant were competed in LB medium with or without bile salts for 8 h, and the log_10_ competitive index (wild type/mutant ratio) was calculated. Error bars represent the standard error of means of the competitive index. *, *P* < 0.05; **, *P* < 0.01.

We determined that E. coli MG1655 Str^r^ Δ*ompC*::*cam* is sensitive to bile salts at concentrations (0.1, 0.25, and 0.5%) typically present in the intestine (Fig. 7A to C). While E. coli MG1655 Str^r^ Δ*ompC*::*cam* growth is comparable to that of the wild type in LB medium in the absence of bile salts (Fig. S7A), at a 0.1% bile salt concentration in LB medium, E. coli MG1655 Str^r^ Δ*ompC*::*cam* has a slightly longer lag phase and slightly lower growth rate during the exponential phase than the wild type (Fig. 7A). At 0.25 and 0.5% bile salt concentrations, E. coli MG1655 Str^r^ Δ*ompC*::*cam* lags for more than 6 h before it enters exponential phase (Fig. 7B and C). At a higher bile salt concentration (1%), E. coli MG1655 Str^r^ Δ*ompF*::*cam* grows slightly faster than the wild type (Fig. S7B). E. coli MG1655 Str^r^ Δ*ompC*::*cam* fails to grow in LB medium containing 5% bile salts (data not shown).

Next, we competed E. coli MG1655 Str^r^ Δ*ompC*::*cam* with the E. coli MG1655 Str^r^ Nal^r^ wild type in LB, LB containing 0.1% and 0.2% bile salts for 8 h. E. coli MG1655 Str^r^ Δ*ompC*::*cam* grew equally well with the wild type in LB medium without bile salts, but as the bile salt concentration was increased, E. coli MG1655 Str^r^ Δ*ompC*::*cam* was outcompeted by the wild type, as indicated by the log_10_ competitive index (wild type/mutant ratio) values of 1.17 and 3.78, respectively, in the presence of 0.1% and 0.2% bile salts (Fig. 7D). The difference in competitive index values between LB medium and LB plus 0.1% bile is statistically significant (*P* = 0.027). Similarly, the difference in competitive index values between LB and LB plus 0.2% bile (*P* = 0.009) and between LB plus 0.1% bile and LB plus 0.2% bile (*P* = 0.008) is statistically significant.

### Bile salt tolerance is important for colonization.

**FIG 8:**
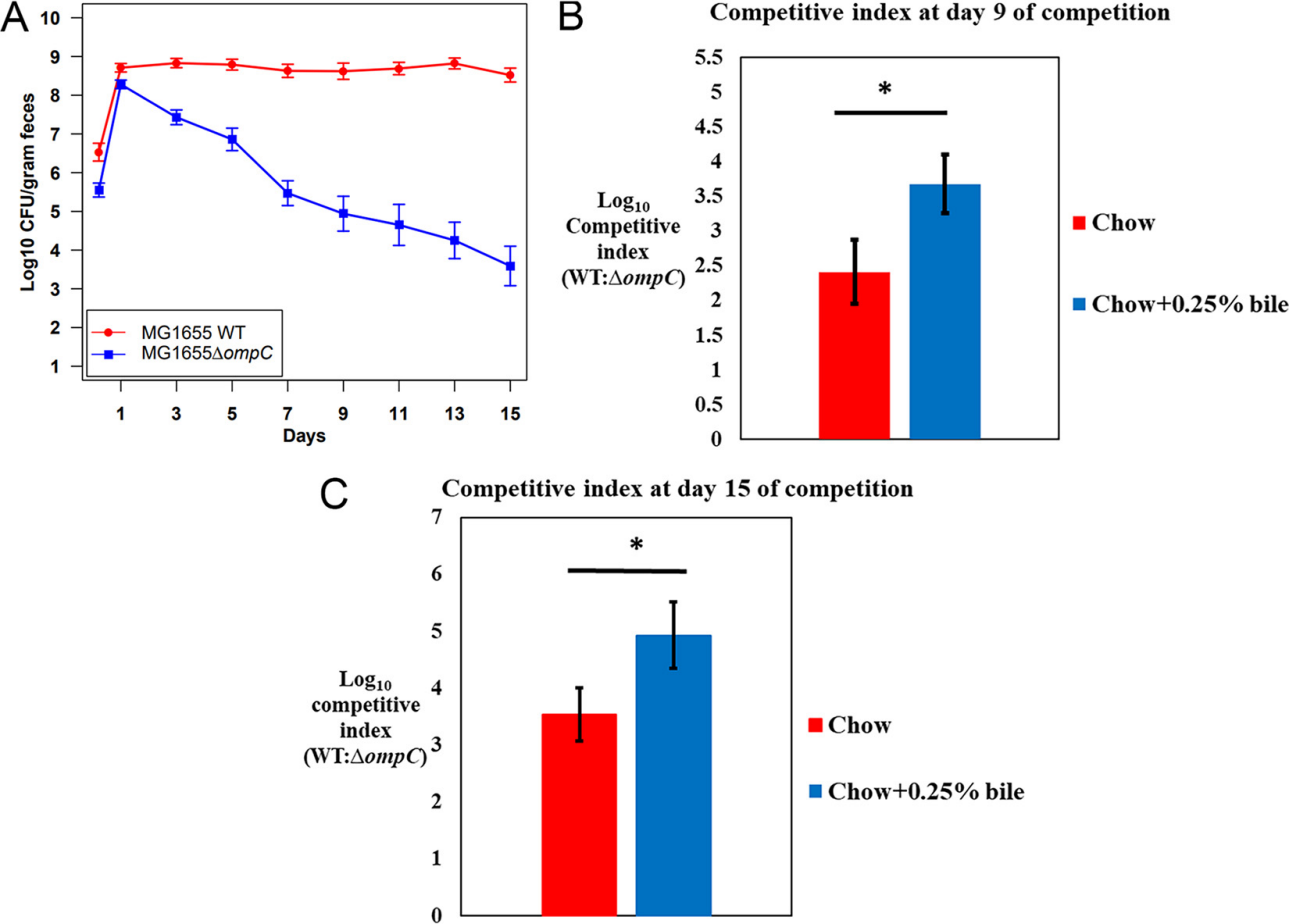
Colonization of the mouse intestine by the E. coli MG1655 Str^r^ Nal^r^ wild type and E. coli MG1655 Str^r^ Δ*ompC*::*cam* mutant when mice were fed with chow containing bile salts. (A) Four sets of three CD-1 male mice, which were fed with standard chow containing 0.25% bile salts, were fed 10^5^ CFU of the E. coli MG1655 Str^r^ Nal^r^ wild type and 10^5^ CFU of the E. coli MG1655 Str^r^ Δ*ompC*::*cam* mutant. (B) Log_10_ competitive index (WT/mutant ratio) in mice fed with chow containing 0.25% bile salts (9A) compared to that of mice fed with standard chow at day 9 of competition (Fig. 2B and D); (C) log_10_ competitive index (WT/mutant ratio) in mice fed with chow containing 0.25% bile salts (9A) compared to that of mice fed with standard chow at day 15 of competition (Fig. 2B). *, *P* < 0.05.

Since E. coli MG1655 Str^r^ Δ*ompC*::*cam* grew more slowly and competed poorly with the wild type *in vitro*, we hypothesized that physiological concentration of bile salts affects E. coli MG1655 Str^r^ Δ*ompC*::*cam* colonization of the intestine. Feeding bile to mice increases the concentration of bile in the duodenum and cecum (26). If bile salts are responsible for E. coli MG1655 Str^r^ Δ*ompC*::*cam* colonizing poorly during competition against the wild type, feeding food mixed with bile salts to mice should exacerbate the colonization defect. So, we fed chow mixed with 0.25% bile salts to mice during competition of the wild type and E. coli MG1655 Str^r^ Δ*ompC*::*cam*. E. coli MG1655 Str^r^ Δ*ompC*::*cam* was outcompeted faster and to a larger extent in mice fed chow mixed with bile salts compared to standard chow (Fig. 2B and Fig. 8A, B, and C). By day 9 of competition, the log_10_ competitive index (WT/mutant ratio) was 3.67 when mice were fed chow containing 0.25% bile salts compared to 2.41 when fed standard chow (*P* = 0.016). Similarly, by day 15 of competition, the log_10_ competitive index (WT/mutant) was 4.93 when fed chow containing 0.25% bile compared to 3.53 when fed standard chow, which again is statistically significant (*P* = 0.044). It is also clear (Fig. 2B and Fig. 8A) that E. coli MG1655 Str^r^ Δ*ompC*::*cam* is outcompeted faster in the presence of excess bile salts. This indicates that E. coli MG1655 Str^r^ Δ*ompC*::*cam* is outcompeted by the wild type due to the presence of bile salts in the intestine, which apparently enter the E. coli periplasm through OmpF.

### RNA-seq analysis reveals activation of the EnvZ/OmpR two-component system, upregulation of OmpC, and downregulation of OmpF.

**FIG 9:**
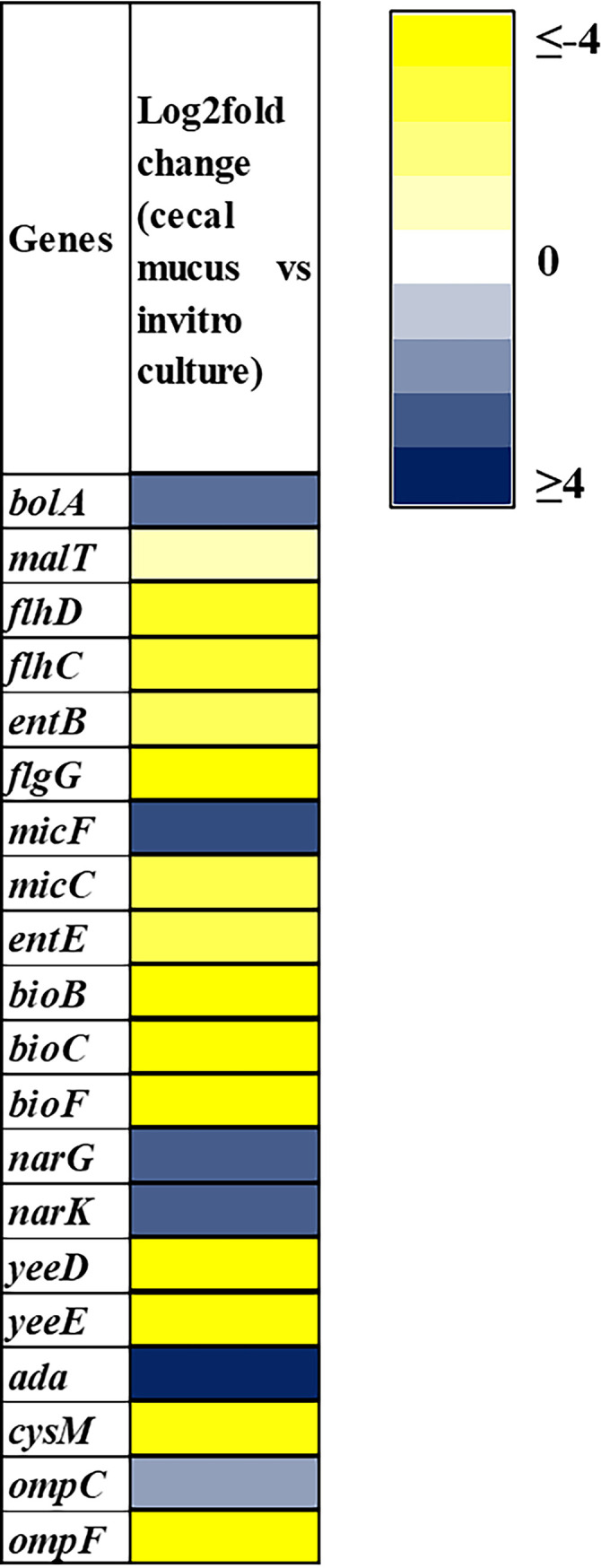
Heat map showing E. coli genes upregulated or downregulated in mouse cecal mucus compared to *in vitro* log-phase culture in MOPS medium (log_2_ fold).

To investigate control of the EnvZ/OmpR regulon in the intestine, we sequenced RNA extracted from cecal mucus in mice colonized with E. coli and compared gene expression with log-phase RNA extracted from E. coli grown on MOPS minimal glucose medium. The results showed that genes under positive regulation by the EnvZ/OmpR two-component system were induced and genes under negative control were downregulated *in* E. coli in the intestine (6). This finding is expected since the concentration of salt in the intestine exceeds the level required to stimulate EnvZ (27). Some representative genes regulated by the EnvZ/OmpR two-component system that are differentially regulated in the mouse intestine are shown in Fig. 9. The results confirm our finding that the EnvZ/OmpR two-component system is active during colonization of the intestine. Furthermore, in E. coli growing in the intestine, the *ompC* gene is upregulated log_2_ 1.73-fold, whereas the *ompF* gene is downregulated log_2_ 5.56-fold (Fig. 9). *micC*, which is a small-RNA negative regulator of *ompC* (28) is downregulated log_2_ 2.79-fold, and *micF*, which is a small-RNA negative regulator of *ompF* (29), is upregulated by log_2_ 3.22-fold in the mouse intestine, which confirms our finding that the high OmpC/OmpF ratio is crucial for E. coli colonization of the mammalian intestine.

## DISCUSSION

In this study, we showed the EnvZ-OmpR two-component system, two of the three outer membrane proteins (OmpC and OmpA), and bile salt tolerance contribute to colonization of the mammalian intestine, while OmpF is nonessential and deleterious for *E. coli* colonization. Deletion of *ompF* increases production of OmpC, and together these changes provide an advantage for E. coli colonization. Our finding that the EnvZ-OmpR two-component system is important for E. coli colonization corroborates our previous finding (1) that spontaneous *envZ* missense mutants colonized the mouse intestine better than the wild type. Giraud et al. (7) also showed that when wild-type E. coli MG1655 was fed to germfree mice, the intestine selected the better-colonizing E. coli MG1655 *envZ* missense mutants. However, Lasaro et al. (30) showed that OmpR was not important for a mouse isolate, E. coli MP1, to colonize the mouse intestine, suggesting the importance of the system could be strain specific.

More than 100 genes are regulated by EnvZ/OmpR, including genes involved in amino acid metabolism (cysteine and isoleucine biosynthesis), enterochelin biosynthesis, and flagellar biosynthesis, as well as those that encode the outer membrane proteins OmpC and OmpF (6). Since the better-colonizing *envZ* mutants had different outer membrane protein levels from the wild type (5, 7), we constructed *ompA*, *ompC*, and *ompF* knockout mutants. During competition with the wild type, the Δ*ompA*::*cam* and Δ*ompC*::*cam* mutants were outcompeted by the wild type. Strikingly, the Δ*ompF*::*cam* mutant colonized better than the wild type by over log_10_ 2-fold during 15 days of competition. Since OmpF has a slightly larger pore size than OmpC, larger molecules, including toxic bile salts, can diffuse into the periplasm. This explains why E. coli preferentially expresses OmpC and downregulates OmpF in the presence of bile salts (10, 14, 15). We demonstrated previously that the better-colonizing *envZ* mutants had higher levels of OmpC and lower levels of OmpF than the wild type, confirming our finding that OmpC is important for adaptation to the intestine while OmpF is deleterious (5, 7).

According to Freter’s nutrient-niche hypothesis, different species of bacteria can coexist in the intestine when each one grows better than all others on at least one limiting nutrient (31). Wild-type E. coli persists in the intestine of *ompC* mutant-precolonized mice, suggesting the wild type might grow better on at least one limiting nutrient. However, the wild type and *ompC* mutant grew equally well on 9 different sugars, suggesting that there is another unknown niche that is not saturated by the *ompC* mutant. It is also possible that the niche is not nutritional; since OmpC is known to be an adhesin (32) and may increase the binding affinity to attachment sites on a mixed biofilm (5), the *ompC* mutant might fail to saturate a physical niche. We also showed here that *ompA* and *ompF* mutants compete in the same niche as the wild type in the intestine, meaning the mutants do not have a nutritional advantage. These findings support the idea that in addition to occupation of nutrient-defined niches E. coli must also tolerate environmental stressors. This can be explained by the “restaurant” hypothesis of colonization (33, 34); the *ompA and ompF* mutants occupy the same restaurant as the wild type and display the same nutritional programs in the intestine (5) but possibly differ in their tolerance to environmental stressors in those restaurants.

The Δ*ompF*::*cam* mutant, which outcompetes the wild type, lacked OmpF and produced more OmpC, which contributed to superior colonization. Since OmpC and OmpF are reciprocally regulated by EnvZ/OmpR and the total amount of the two proteins remains constant, reduction in the level of one protein is compensated for by an increase in the other (17, 19). Our findings are consistent with the findings of Schnaitman and McDonald (20), where deletion of *ompC* led to constitutive expression of OmpF protein. Ozawa and Mizushima (21) also showed that OmpF expression regulates the transcription of *ompC*, which is in agreement with our findings. The importance of OmpC and deleterious role of OmpF are confirmed by our *in vivo* competition between the Δ*ompC*::*kan* and Δ*ompF*::*cam* mutants, in which *the* Δ*ompF*::*cam* mutant outcompeted the Δ*ompC*::*kan* mutant by nearly log_10_ 4-fold. We showed previously that better-colonizing *envZ* mutants produced higher OmpC, while OmpF was reduced (5, 7). However, higher OmpC alone is not sufficient for better colonization and requires both overproduction of OmpC and deletion of *ompF*, which indicates that regulation of OmpC and OmpF by EnvZ/OmpR is fine-tuned to maximize the competitiveness of E. coli in the intestine. Sequencing of RNA from colonized E. coli demonstrated that genes activated by EnvZ/OmpR, including *ompC*, were upregulated and genes repressed by EnvZ/OmpR, including *ompF*, were downregulated in the intestine.

Why is OmpC important for E. coli during colonization? We showed that the E. coli MG1655 Str^r^ Δ*ompC*::*cam* mutant adapts slowly to conditions in the intestine, with limited growth during the first 15 h, suggesting that it enters its mucosal niche later than the wild type. Since E. coli thrives in mixed biofilms in the mucus layer of the intestine (1) and OmpC functions as an adhesin (32), it is possible that E. coli MG1655 Str^r^ Δ*ompC*::*cam* faces adverse factors in the intestinal lumen for a longer period of time. Indeed, E. coli MG1655 Str^r^ Δ*ompC*::*cam* is sensitive to physiological bile salt concentrations present in the intestine. This is due to the presence of OmpF, which allows bile salts to penetrate the periplasm. Although enteric bacteria have efflux systems such as AcrAB-TolC to efflux the bile salts from the cytoplasm and a complex regulatory system, including the *marRAB* operon, *soxS*, and *robA* to regulate the efflux (35, 36), efflux systems are energy dependent (37). As energy efficiency contributes to competitive fitness of E. coli in the mouse intestine (38), it may be advantageous for E. coli to differentially regulate outer membrane proteins to block entry of bile salts into the cell rather than to efflux them after they enter the cell.

It is also possible that other unknown factors besides exclusion of bile salts contribute to the importance of OmpC in the intestine. It has been reported previously that E. coli increases iron uptake using the OmpC-FeoB-mediated Fe^2+^ transport pathway (39), suggesting that OmpC might be important for iron uptake in the intestine. Although not known, gut microbiota-derived metabolites or toxic substances could also differentially diffuse through OmpC and OmpF. Lactobacilli administered together with oligosaccharides reduced adherence and colon counts of E. coli, which could be due to increased acetic acid production in the gut (40). However, the role of outer membrane proteins in organic acid uptake has not been explored. OmpC is also an adhesin (32), which could allow better adherence to attachment sites in the mixed biofilms. Alternatively, besides allowing bile salts to pass through, OmpF could also be disadvantageous because it is a receptor for colicins and phages (41–45). Bdellovibrio bacteriovorus, present in the gut of animals and healthy humans (46), uses OmpF to recognize and attack *E. coli* (47). However, it is unlikely that an obligate aerobe such as *Bdellovibrio* predominates in the streptomycin-treated mouse gut.

It is also known that EnvZ/OmpR responds to environmental signals, such as pH and osmolarity, and regulates the expression of outer membrane porins OmpC and OmpF (19, 48). When we grew the E. coli MG1655 WT, Δ*ompC* mutant, and Δ*ompF* mutant strains in LB medium containing 0.3 M NaCl (which is equivalent to the osmolarity of the gut lumen), all three strains grew equally well, suggesting that osmolarity of the intestine is not inhibitory to a Δ*ompC* mutant (unpublished work). *Salmonella* Typhi Δ*ompR*, Δ*ompC*, and Δ*ompC* Δ*ompF* mutants, when grown on LB medium with 0.3 M NaCl, also grew at the same rate as the wild type (16). Acid resistance of Δ*ompC*, Δ*ompF*, and Δ*ompC* Δ*ompF* E. coli mutants was slightly reduced compared to that of wild-type E. coli, as indicated by their reduced viability when grown at a pH 5.5 and then subsequently challenged by extremely low pH (2.5 for 1 h) (49). The study reported that even the wild-type survival rate was around 1%. Our model system is different in that we feed overnight cultures (stationary phase) of E. coli. E. coli has developed at least 5 different acid resistance (AR) systems responsive to extreme acid stress and an acid tolerance response (ATR) system in response to mild and moderate acid stress (50). These systems can protect stationary-phase cells from extreme acid stress, and mammals almost always ingest E. coli cells in their stationary phase, which already have acid resistance systems induced. Because of these extensive systems in response to acid stress, E. coli can tolerate pH 2 for several hours without growing (50, 51). If only 1% of cells survive on exposure to stomach pH, as demonstrated by Bekhit et al. (49), this cannot explain the almost 1.5-fold log increase in wild-type E. coli recovered at 5 h postfeeding compared to the population of wild-type cells fed to mice in our experiments (Table 1). These reports suggest that pH and osmolarity may not explain the importance of OmpC in the intestine.

In summary, our findings clearly establish the role of OmpC in colonization of the mammalian intestine by E. coli. OmpC is essential for bile salt tolerance and hence colonization, while OmpF allows bile salts to enter the periplasm and is deleterious for colonization. Failure to properly regulate Omp expression results in a lag during initiation of colonization. Thus, EnvZ/OmpR-dependent upregulation of *ompC* and downregulation of *ompF* are critical during colonization of the mammalian intestine.

## MATERIALS AND METHODS

### Bacterial strains and plasmids.

The bacterial strains and plasmids used in this study are listed in Table 2. The original *E coli* strain K-12 was isolated in Palo Alto, CA, in 1922 from a stool sample from a patient recovering from diphtheria (52). The sequenced E. coli MG1655 strain used in this study was derived from the original K-12 strain by curing the temperate bacteriophage lambda by means of UV light and F plasmid by acridine orange treatment (5).

**TABLE 2:** *E. coli* strains and plasmids used in the study

Strain or plasmid	Description	Reference
Strains		
MG1655 Str^r^	Spontaneous streptomycin-resistant mutant of MG1655	1
MG1655 Str^r^ Nal^r^	Spontaneous nalidixic acid-resistant mutant of MG1655 Str^r^	1
MG1655 Str^r^ Δ(*envZ-ompR*)::*cam*	1,965-bp deletion in (*envZ-ompR*) genes in *ompB* locus replaced by chloramphenicol resistance cassette	This study
MG1655 Str^r^ Δ*ompA*::*cam*	937-bp deletion in *ompA* gene replaced by chloramphenicol resistance cassette	This study
MG1655 Str^r^ Δ*ompC*::*cam*	1,000-bp deletion in *ompC* gene replaced by chloramphenicol resistance cassette	This study
MG1655 Str^r^ Δ*ompC*::*kan*	1,000-bp deletion in *ompC* gene replaced by kanamycin resistance cassette	This study
MG1655 Str^r^ Δ*ompF*::*cam*	1,012-bp deletion in *ompF* gene replaced by chloramphenicol resistance cassette	This study
MG1655 Str^r^ Cam^r^ Δ*ompC* Δ*ompF*	Deletions in *ompC* and *ompF* genes	This study
MG1655 Str^r^ P*_trc_*::*ompC*	*ompC* placed under control of trc promoter and OmpR binding sites upstream of *ompC* and its native promoter deleted	This study
MG1655 Str^r^ Cam^r^ P*_trc_*::*ompC* Δ*ompF*	*ompC* placed under control of trc promoter, OmpR binding sites upstream of *ompC* and its native promoter deleted, *ompF* gene deleted and replaced by chloramphenicol cassette	This study
Plasmids		
pKD3	Template plasmid for *frt*-flanked chloramphenicol acetyltransferase cassette	53
pKD4	Template plasmid for *frt*-flanked kanamycin cassette	53
pCP20	Helper plasmid, which expresses FLP recombinase.	53
pKD267	Plasmid contains *parE* under control of rhamnose promoter and kanamycin resistance gene	Gift from Barry L. Wanner

### Construction of mutants.

Mutants were constructed by using the allelic exchange method described previously by Datsenko and Wanner (53). The gene of interest was deleted and replaced by either a chloramphenicol or kanamycin resistance cassette. Multiple-gene deletion mutants, when needed, were constructed by removing the first antibiotic resistance cassette with FLP recombinase. The second gene deletion was made by sequential allelic replacement and confirmed by PCR using primers specific to the gene and cassette sequences. To construct the strain constitutively overexpressing OmpC, a markerless allelic replacement method developed by Wanner and Datsenko was used as described previously (1). First, the regulatory region (OmpR binding sites upstream of promoter) and the native promoter of *ompC* were replaced by a cassette encoding kanamycin resistance (*kan*) and ParE toxin under the control of a rhamnose-inducible promoter (*kan-rhaP*::*parE*). Kanamycin-resistant isolates were selected on LB plates containing kanamycin and confirmed by PCR using primers specific for the deleted sequence and the cassette. In the second step, the *kan-rhaP*::*parE* cassette was replaced with a *trc* promoter (P*_trc_*) using the allelic replacement strategy described by Wanner (53). The construct was selected by growing on M9 minimal agar plates containing 1% rhamnose as the sole source of carbon, which would induce *parE*, if present. The *parE* toxin gene encodes a DNA gyrase inhibitor, and cells retaining the cassette die on M9 plates with rhamnose. The constructed strain was confirmed by PCR and sequencing for the loss of the native promoter and replacement by the *trc* promoter. Constitutive overexpression of OmpC was determined by outer membrane protein analysis as described previously (5).

### Mouse colonization experiments.

All mouse experiments were conducted following the ethical guidelines outlined in the *Guide for the Care and Use of Laboratory Animals* by NIH (54), and the protocol was approved by Oklahoma State University IACUC (ACUP no. 19-61). Mouse colonization experiments were performed as previously described (1, 5, 55–57). Briefly, at least two sets of three CD-1 male mice 6 to 8 weeks old (Charles River Laboratories, Wilmington, MA) were acclimated for 5 days following delivery. One day prior to the experiment, the mice were transferred to individual cages and given water containing streptomycin sulfate (5 g/L) for 24 h to eliminate resident facultative anaerobes. Next, the mice were starved for food and water for 18 h. The mice were then fed 1 mL of 5% sucrose solution containing 10^5^ CFU of one or more E. coli strains (day 0). Following consumption of the bacterial suspension (no longer than 1 h), food and streptomycin-containing water were returned to the mice (*ad libitum*). At 5 h, 24 h, and on odd-numbered days thereafter for 15 days, fecal samples were collected, homogenized in 1% Bacto tryptone, serially diluted in 1% Bacto tryptone, and plated on MacConkey agar plates containing appropriate antibiotics. If the number of CFU per gram of feces of any strain was expected to be below 10^2^, 1 mL of fecal suspension was centrifuged at 12,000 × *g* for 5 min, the supernatant was discarded, and then the pellet was resuspended in 100 μL of 1% Bacto-tryptone and plated on appropriate MacConkey plates. To ensure that the fecal pellets were not older than 24 h, cages were changed on even-numbered days. Plates were incubated at 37°C for 24 to 48 h before counting the colonies. The log_10_ CFU per gram of feces was determined for each time point for each strain from individual mice, and the average CFU and standard error were reported. Data from at least two independent experiments were pooled (≥6 mice total). A Student's *t* test was used to determine significance. A difference of ≥10^1^ CFU/g feces between strain populations in all cases was statistically significant (*P* < 0.05).

For challenge experiments, on the 9th day, the mice were again starved for 18 h, and on the 10th day, the mice were fed ~10^5^ CFU of the challenge E. coli strain in 1 mL of 5% sucrose solution. Food and water were returned to mice, and the fecal samples were collected at 5 h, at 24 h, and on odd-numbered days until day 21. Samples were processed and cages were changed as described above.

To study the effect of bile salts on colonization, mice were fed irradiated chow containing 0.25% bile salts (bile salts no. 3, catalog no. B11750-100.0; Research Products International, Mt. Prospect, IL) from 2 days prior to beginning the experiment until the experiment was completed as described previously (58). Briefly, the pelleted feed was ground into a powder and then mixed with the appropriate weight of bile salts using a mixer. The mice were then provided with feed containing bile salts in glass bowls. The glass bowls were changed on days 5 and 12 to minimize contamination with feces and urine.

### Outer membrane protein analysis.

Outer membrane protein analysis was carried out as previously described (5, 59). Briefly, E. coli strains were grown overnight on LB broth containing 100 μg/mL of streptomycin sulfate with shaking at 37°C. The cells were then diluted to an *A*_600_ of ~0.1 in low-osmolarity medium (medium A) (48) and grown to an *A*_600_ of 0.6 to 0.9. Thirty milliliters of each culture was centrifuged at 3,000 × *g* for 15 min at 4°C, and the pellets were kept at −80°C until they were processed. The cell pellets were thawed on ice and lysed by treatment with lysozyme (50 μL of 5 mg/mL in 5 mM Tris-HCl [pH 8.0] plus 0.1 M EDTA [pH 8.0] for 30 min in ice). After incubation, 5 mL of cold 3 mM EDTA was added and mixed by vortexing. Then samples were sonicated three times for 30 s using a 550 Sonic Dismembrator (Fisher Scientific) at a setting of 4, and membrane pellets were isolated by high-speed centrifugation, as described previously (5, 59), and then suspended in 100 μL of Tris-HCl (pH 8.0). The protein concentration in each sample was estimated by using the Bradford assay (60). Equal amounts of each protein sample were suspended in 4× Laemmli buffer and heated at 95°C for 10 min. Electrophoresis was conducted on 10% SDS–polyacrylamide gels containing 4 M urea for 2 to 3 h at 125 V. Three independent outer membrane protein analysis experiments were run. The densitometric analysis of the bands in the gels was conducted by using ImageJ (version 1.53t) as described previously (61).

### Bradford assay.

The Bradford assay to estimate the concentration of protein was accomplished as described previously, with a few modifications (60). Briefly, bovine serum albumin (BSA) dilutions containing 100 to 1,000 μg/mL protein were prepared from a protein standard of 2.0 mg/mL (Thermo Fisher Scientific; catalog no. 23209) by dilution in Tris-HCl (pH 8.0). Then, 20 μL of each diluted BSA sample was mixed with 1 mL of Bradford reagent (Thermo Fisher Scientific; catalog no. 23238) in a microcentrifuge tube and incubated at room temperature for 5 min. The mixture was transferred to a cuvette, and the *A*_595_ was measured against the reagent blank prepared by mixing 20 μL of Tris-HCl (pH 8.0) and 1 mL of Bradford reagent. All of the measurements were carried out in duplicate, and the protein concentration was plotted against the average absorbance to generate the standard curve. The standard curve was then used to calculate the protein concentration in samples.

### Bile sensitivity testing.

Bile salt sensitivity testing of different E. coli strains was performed as described previously by Leatham-Jensen et al. (1), with few modifications. Different E. coli strains were grown overnight on LB broth supplemented with 100 μg/mL of streptomycin sulfate with shaking at 37°C. Overnight cultures were diluted 1:100-fold in LB containing different concentrations of bile salts no. 3. One hundred microliters of diluted culture was pipetted into 96-well microtiter plates, and growth at 37°C was monitored spectrophotometrically every 15 min with a microplate reader (Bio Tek Synergy H1). The experiment was repeated in triplicate.

### Extraction of bacterial RNA from mouse cecal mucus.

A modified protocol for RNA isolation from mouse cecal mucus was developed based on the methods published by M. Ares (62) and Donaldson et al. (63). Streptomycin-treated mice colonized with E. coli were euthanized by CO_2_ asphyxiation, followed by cervical dislocation. Immediately, their abdomen was cut open and the cecum was removed. The luminal contents were squeezed out, and then the lumen was washed gently with 1 to 2 mL of Hanks salt solution (Thermo Scientific; catalog no. 88284) to remove residual feces. Then, 500 μL of Hanks salt solution was pipetted into the cecal sac and gently massaged to dislodge the mucus, and the mixture was poured into a Lysis Matrix B tube (MP Biochemicals; catalog no. 116911100) containing 500 μL of PCA (phenol-chloroform-isoamyl alcohol at 25:24:1) and 210 μL of 20% SDS. The cells were lysed by bead beating for 70 s using a Bead Bug microtube compact homogenizer at 4,000 rpm (Benchmark Scientific; model no. 1030E), and the tubes were kept on ice until further processing. All steps in the protocol were completed within 5 min.

The samples were centrifuged at 13,000 rpm for 3 min at 4°C, and the aqueous phase was carefully transferred to a new microcentrifuge tube containing 500 μL of PCA and mixed well. The tube was centrifuged at 13,000 rpm for 3 min at 4°C, and the aqueous phase was transferred to a Phase Lock Gel tube (Quantabio, Beverly, MA; catalog no. 2302830) containing 500 μL of PCA and mixed gently by inversion. The centrifugation was repeated, leaving everything in the tube, and 250 μL chloroform was added, mixed by inversion, and centrifuged again. This step of mixing with chloroform and centrifugation was repeated twice in succession. Finally, the aqueous phase from 3 samples was pooled and mixed with 50 μL of 3 M sodium acetate (pH 5.2), and 100% isopropanol was added to bring the volume to 2 mL. The tube was mixed by inversion and incubated at −70°C overnight. The next day, the tubes were thawed on ice and centrifuged at maximum speed for 10 min at 4°C, isopropanol was poured off, 1 mL of ice cold 70% ethanol was added, and the mixture was incubated on ice for 15 min. The tube was again centrifuged for 10 min at 4°C, the ethanol was carefully poured off, and the pellet was allowed to air dry. The dried pellet was dissolved in 80 μL of RNase/DNase-free water by vortexing for 5 min. The nucleic acids were quantified using an Eppendorf BioPhotometer.

To prepare DNA-free RNA, 10 μL of DNase I buffer and 10 μL of DNase I enzyme (Invitrogen; catalog no. AM2238) were added to the RNA sample as described previously (64). The mixture was incubated at 37°C for 30 min, and then the DNase was inactivated by adding 10 μL of 0.5 M EDTA, (pH 8.0) (Invitrogen, Waltham, MA; catalog no. 15575-038) and heating at 65°C for 10 min. Water was added to bring the final volume to 200 μL, 200 μL of PCA was added for phenol-chloroform extraction, and the RNA was precipitated with ethanol as described above.

### Extraction of bacterial RNA from *in vitro* cultures.

Morpholinepropanesulfonic acid (MOPS) minimal medium, prepared as described previously (65), was used to culture bacteria for RNA extraction. E. coli MG1655 Str^r^ Nal^r^ was grown overnight on 5 mL of MOPS medium containing 0.05% glucose at 37°C and shaking at 200 rpm. Fifty milliliters of MOPS medium containing 0.2% glucose was inoculated with 50 μL of the overnight culture. Cultures were harvested at an *A*_600_ of ~0.4 to 0.6 for exponential-phase bacteria by mixing 5 mL of culture with 10 mL of RNAprotect reagent (Qiagen; catalog no. 76506). After 5 min of incubation, the cells were pelleted, and RNA was extracted using a RNeasy minikit (Qiagen; catalog no. 74104) according to the manufacturer’s instructions.

### RNA-seq and analysis.

RNA samples were shipped to the University of Bern, Switzerland, Next Generation Sequencing Platform. The samples were analyzed for RNA integrity and quantified using an Agilent Bioanalyzer (Agilent, Santa Clara, CA; catalog no. G2939BA). Approximately 100 ng of total RNA from each sample was processed for ribo-depletion with the Illumina Ribo-Zero Plus rRNA depletion kit (Illumina, San Diego, CA; catalog no. 20037135). Ribo-depleted RNA samples were prepared for sequencing using the TruSeq stranded total RNA library prep kit. Libraries were sequenced on a NovaSeq 6000 system for 1 × 100-bp single-end reads.

Sequence reads were mapped to the E. coli MG1655 reference genome (RefSeq accession no. U00096.3) using bowtie2 (66). SAM files from the bowtie2 alignment were converted to BAM files and then to BigWig files using SAMtools (67). Mapped reads were visualized in Jbrowse (68). The approach used for data analysis was described previously (69).

### *In vitro* growth competition assay.

*In vitro* competition assays were performed as described previously (70), with a few modifications. Briefly, E. coli MG1655 Str^r^ Nal^r^ and E. coli MG1655 Str^r^ Δ*ompC*::*cam* were grown overnight on LB medium at 37°C. Ten microliters from overnight cultures of each strain was added to 980 μL of LB in a microcentrifuge tube (1:100 dilution). Then, 100 μL of the mixed culture was added to a flask containing 10 mL of LB, 10 mL of LB containing 0.1% bile salts, or 10 mL of LB containing 0.2% bile salts and mixed well. To estimate the input CFU for each strain, 1 mL of culture was removed from the conical flask, serially diluted in 1% Bacto tryptone, and plated on MacConkey plates containing streptomycin and nalidixic acid (for wild type) and MacConkey plates containing streptomycin and chloramphenicol (for the mutant). The population of each strain was ~10^5^ CFU/mL at the beginning of the experiment. The flasks were incubated at 37°C with shaking at 200 rpm. After 8 h of growth, 1 mL of culture was removed from the flasks and serially diluted and plated as described above to estimate the relative growth of each strain. The competitive index was calculated by using the following formula (71): (CFU of wild type at 8-h time point/CFU of mutant at 8-h time point)/(CFU of wild type inoculated at 0-h time point/CFU of mutant inoculated at 0-h time point).

The experiment was repeated in triplicate.

### Carbon source utilization.

Carbon source utilization by E. coli MG1655 Str^r^ Nal^r^, E. coli MG1655 Str^r^ Δ*ompC*::*cam*, and E. coli MG1655 Str^r^ Δ*ompF*::*cam* was determined in MOPS minimal medium supplemented with arabinose (0.2% [wt/vol]), fructose (0.2% [wt/vol]), galactose (0.2% [wt/vol]), gluconate (0.2% [wt/vol]), glucose (0.2% [wt/vol]), maltose (0.2% [wt/vol]), mannose (0.2% [wt/vol]), *N*-acetylglucosamine (0.2% [wt/vol]), or ribose (0.2% [wt/vol]). The strains were grown overnight on 5 mL of MOPS medium containing 0.05% glucose at 37°C with shaking at 200 rpm. Overnight cultures were then diluted into MOPS medium supplemented with individual carbon sources (sugar) to an optical density at 600 nm (OD_600_) of ~0.01. One hundred microliters of diluted culture was pipetted into 96-well microtiter plates, and growth at 37°C was monitored spectrophotometrically every 15 min with a microplate reader (Bio Tek Synergy H1). The experiment was repeated in triplicate.

### Data availability.

The RNA-seq data are available at GEO under accession no. GSE217743.
