# Supplementary material for: OmpC-Dependent Bile Tolerance Contributes to E. coli Colonization of the Mammalian Intestine
Source: Microbiol Spectr. 2023 Apr 4;11(3):e05241-22. doi: 10.1128/spectrum.05241-22 (PMC10269588; doi:10.1128/spectrum.05241-22)
Supplement: Supplemental file 1 — Supplemental material. Download spectrum.05241-22-s0001.pdf, PDF file, 0.7 MB [file spectrum.05241-22-s0001.pdf]

Supplementary information for

OmpC-dependent bile tolerance contributes to *E. coli* colonization of the mammalian intestine

Sudhir Doranga and Tyrrell Conway*
Department of Microbiology and Molecular Genetics, Oklahoma State University, Stillwater, OK 74078

*Corresponding Author:
Tyrrell Conway
Phone: 405-744-6243
Fax: 405-744-6790
e-mail: tconway@okstate.edu

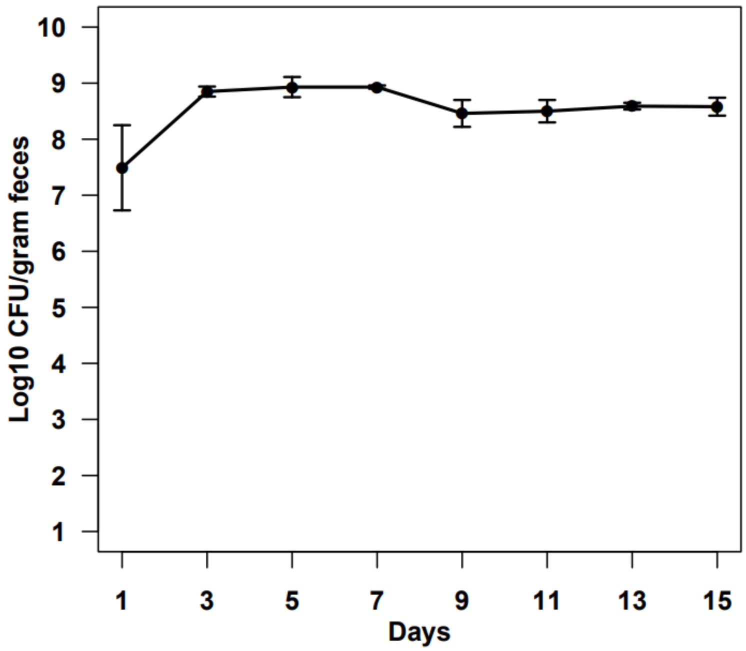

Figure S1. Monocolonization of *E. coli* MG1655 Str^{R} Cam^{R} Δ*(envZ-ompR)* in the mouse intestine. A set of 3 mice were fed 10^5 CFU of *E. coli* MG1655 Str^{R} Cam^{R} Δ*(envZ-ompR)* and fecal samples were collected at 1 day and odd numbered days until the 15th day.

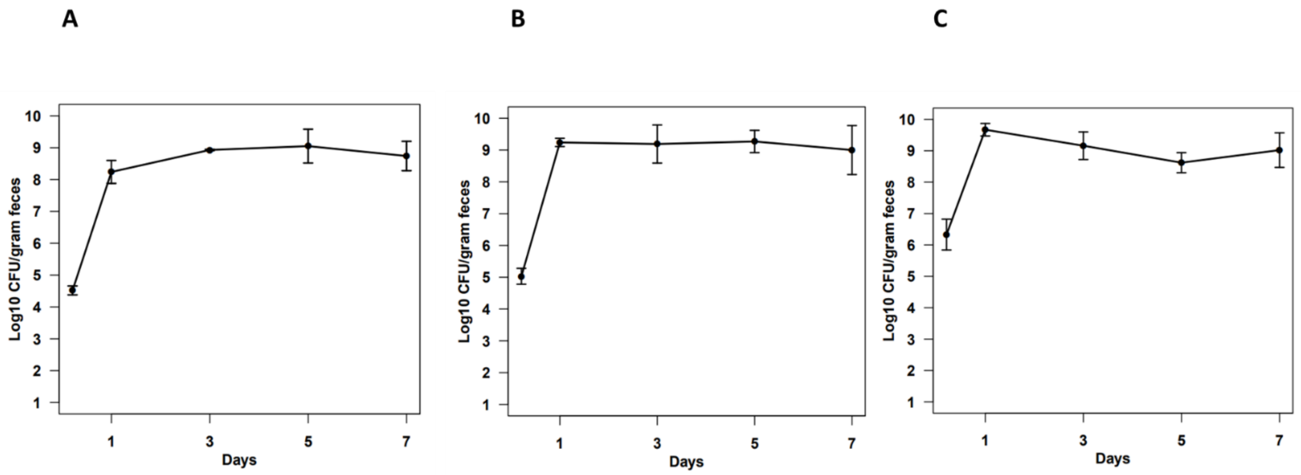

Figure S2: Monocolonization of outer membrane protein mutants in the mouse intestine. (A) A set of 3 mice were fed 10^5 CFU of *E. coli* MG1655 Str^R Δ*ompA::cam* and fecal samples were collected at 5 hours, 1 ,3, 5 and 7 days. (B) A set of 3 mice were fed 10^5 CFU of *E. coli* MG1655 Str^R Δ*ompC::cam* and fecal samples were collected at 5 hours, 1 ,3, 5 and 7 days. (C) A set of 3 mice were fed 10^5 CFU of *E. coli* MG1655 Str^R Δ*ompF::cam* and fecal samples were collected at 5 hours, 1 ,3, 5 and 7 days.

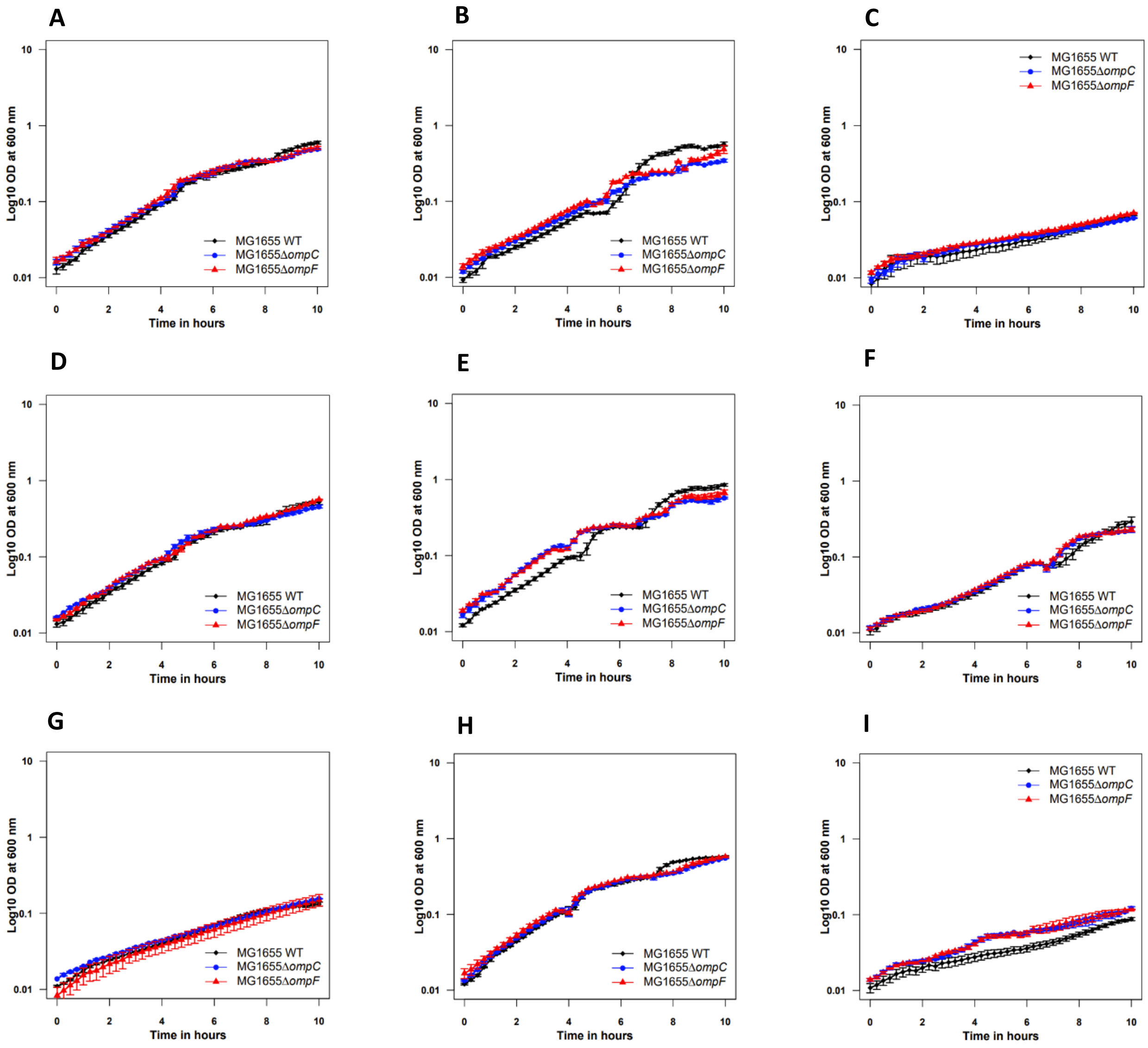

Figure S3. Growth of different *E. coli* strains on MOPS minimal medium containing 0.2% (wt/vol) sugars. *E. coli* MG1655 Str^{R} Nal^{R,} wild type, *E. coli* MG1655 Str^{R} Δ*ompC*::*cam* and *E. coli* MG1655 Str^{R} Δ*ompF*::*cam* were grown on MOPS minimal medium containing 0.2% (wt/vol) sugar: **A)** Arabinose, **B)** Fructose, **C)** Galactose, **D)** Gluconate, **E)** Glucose, **F)** Maltose, **G)** Mannose, **H)** N-acetyl glucosamine, and **I)** Ribose.

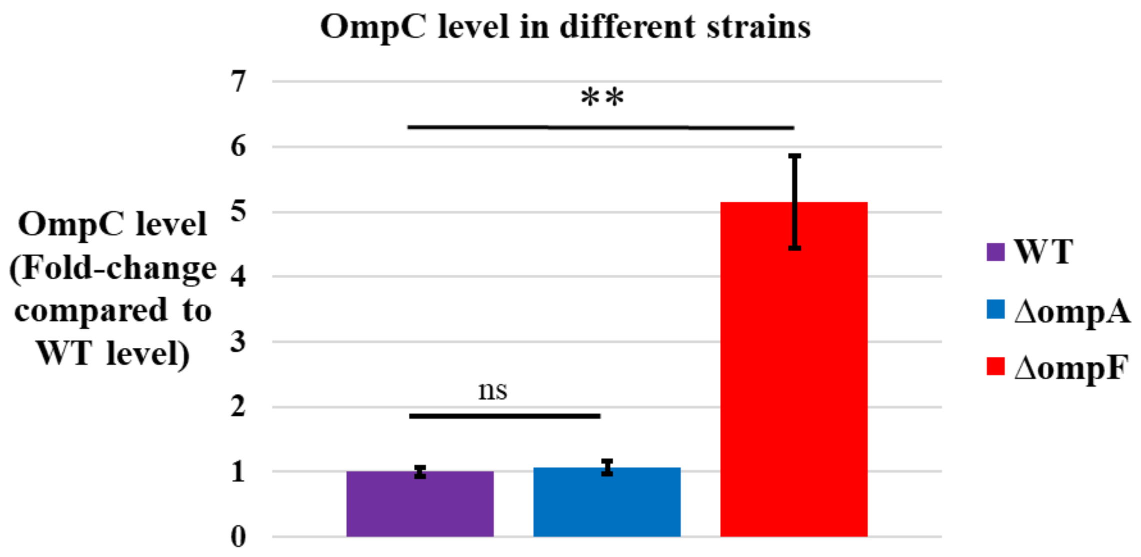

Figure S4. Quantitation of OmpC levels in different strains. OmpC band intensity was quantified and compared to the band intensity of wild type. Y-axis represents fold changes in OmpC levels as compared to OmpC levels in WT. Densitometric analysis is presented as the mean of three experiments. Error bars represent ±S.E.M. t-test for paired samples was used to calculate the statistical differences. ** indicates $p < 0.05$ between paired samples.

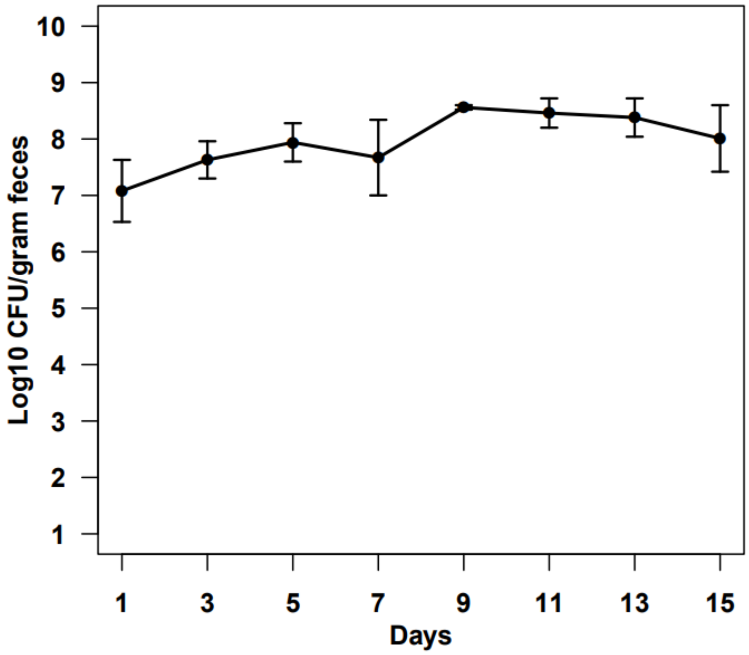

Figure S5. Monocolonization of *E. coli* MG1655 Str^R Cam^R Δ*ompC* Δ*ompF* in the mouse intestine. A set of 3 mice were fed 10^5 CFU of *E. coli* MG1655 Str^R Cam^R Δ*ompC* Δ*ompF* and fecal samples were collected at 1 day and odd numbered days until 15th day.

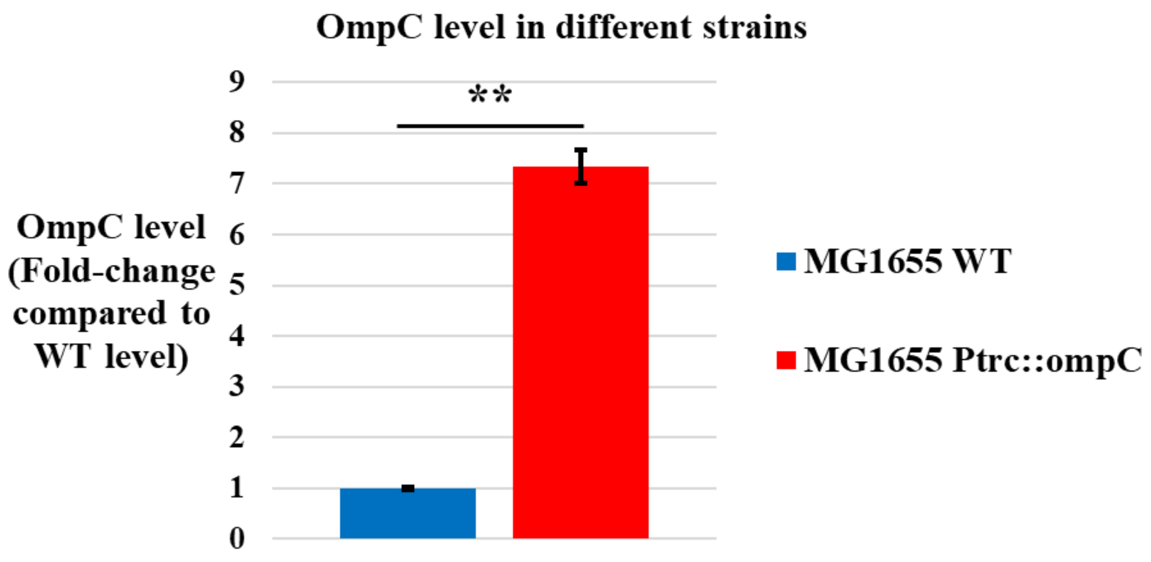

Figure S6. Quantitation of OmpC levels in MG1655 P_{trc}::*ompC*. OmpC band intensity was quantified and compared to the band intensity of wild type. Y-axis represents fold changes in OmpC levels as compared to OmpC levels in WT. Densitometric analysis is presented as the mean of three experiments. Error bars represent ±S.E.M. t-test for paired samples was used to calculate the statistical differences. ** indicates $p < 0.05$ between paired samples.

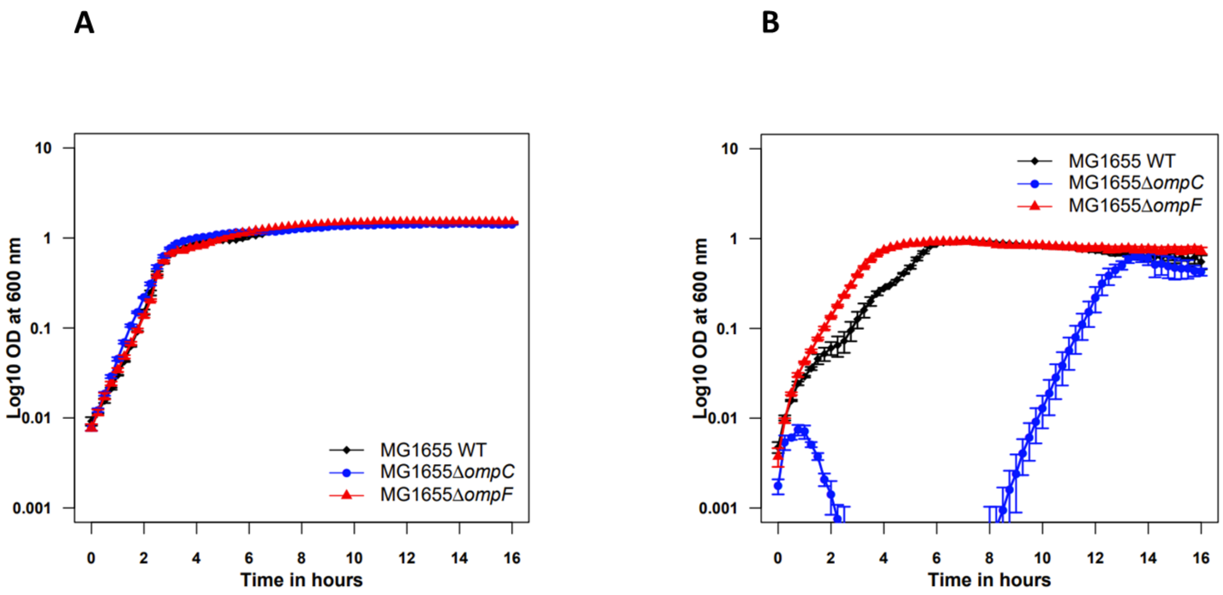

Figure S7. Growth curves of *E. coli* MG1655 Str^{R} Nal^{R} Wild Type, *E. coli* MG1655 Str^{R} Δ*ompC::cam* and *E. coli* MG1655 Str^{R} Δ*ompF::cam* in LB medium with or without bile salts. (A) Growth of *E. coli* strains in LB medium (B) Growth of *E. coli* strains in LB medium containing 1% bile salts.
